# Efficacy of nonsurgical periodontal treatment on patients with periodontitis and type 2 diabetes mellitus: a systematic review and Bayesian network meta-analysis

**DOI:** 10.2340/aos.v84.43344

**Published:** 2025-05-13

**Authors:** Xuejie Xie, Jun Xu, Yiming Li, Li Tang, Gulinuer Awuti

**Affiliations:** Periodontal and Mucosal Department, the First Affiliated Hospital of Xinjiang Medical University (the Affiliated Stomatology Hospital of Xinjiang Medical University), Urumqi, China

**Keywords:** periodontitis, type 2 diabetes mellitus, network meta-analysis, probing depth, clinical attachment loss, bleeding on probing, glycated hemoglobin, fasting blood sugar

## Abstract

**Objective:**

This study aimed to evaluate the effects of periodontal basic therapy combined with various adjunctive treatments on periodontal inflammation and glycemic control in patients with periodontitis and type 2 diabetes mellitus (T2DM) using network meta-analysis.

**Methods:**

Randomized controlled trials (RCTs) involving patients with periodontitis and T2DM were retrieved from PubMed, Embase, Cochrane Library, and Web of Science up to February 29, 2024. The Cochrane quality scoring system was applied to assess study quality, and data were analyzed using R and Stata. The study was registered in PROSPERO (Registration No.: CRD42024501722).

**Results:**

Thirty-seven RCTs involving 1,989 patients were included. Among the adjunctive therapies, scaling and root planing (SRP) with local satranidazole gel (SZ) achieved the best improvement in probing depth (PD) and clinical attachment level (CAL); SRP with systemic amoxicillin (AMX) significantly improved bleeding on probing (BOP); SRP with systemic doxycycline (Doxy) or antimicrobial photodynamic therapy (aPDT) was most effective for reducing glycated hemoglobin (HbA1c%); and SRP with diode laser (DL) improved fasting blood sugar (FBS) most effectively.

**Conclusion:**

SRP combined with local SZ may improve PD and CAL in patients with periodontitis and T2DM. Systemic AMX may enhance BOP outcomes, while DOXY or aPDT may help reduce HbA1c. DL may contribute to better FBS improvement.

## Introduction

Periodontitis is a widely prevalent chronic inflammatory disorder, with dental plaque as the initiating factor. The combined effects of dysbiosis in the periodontal microbiota and the host immune responses can lead to chronic destructive inflammation of the periodontal tissues, ultimately resulting in the destruction of the periodontal supporting tissues and even causing loose tooth and tooth loss [1–3]. Approximately 80% of adults have periodontitis of varying degrees, which is the primary cause of tooth loss in adults [4]. Diabetes is a recognized risk factor for periodontal diseases, while periodontitis is considered the sixth complication of diabetes. Multiple studies have confirmed the bidirectional relationship between periodontitis and type 2 diabetes mellitus (T2DM), with both conditions mutually influencing disease progression and therapeutic outcomes [5–8].

Glycemic control is critical in the treatment of T2DM and is notably associated with the incidence of clinical complications. A 1% reduction in glycated hemoglobin (HbA1c) is linked to a 14% reduction in myocardial infarction risk, a 21% reduction in diabetes-related mortality, and a 37% reduction in microvascular complication risk. Research has demonstrated that scaling and root planing (SRP) or SRP + adjuvant therapies can improve both periodontal conditions and glycemic control in patients with periodontitis and T2DM. However, the optimal therapeutic approach remains inconclusive [9]. Currently, many studies have shown that adjuvant therapies such as the local use of lasers [10, 11], antimicrobial drugs [12], or the systemic use of supplements [13] can significantly improve treatment outcomes for patients with periodontitis and T2DM. However, evidence on the effects of adjuvant therapies on glycemic control given with SRP remains contradictory. For example, compared to SRP alone, topical atorvastatin (ATV) following SRP did not result in additional HbA1c reductions, while adjuvant antimicrobial photodynamic therapy (aPDT) demonstrated additional benefits [14]. Previous meta-analyses primarily compared periodontal treatment with no treatment [15]. Meta-analyses focusing on adjuvant therapies are relatively scarce and are often limited to a single type of intervention, such as aPDT [16] or systemic antibiotics [17, 18]. There is currently no consensus on which adjuvant therapy combined with SRP is most effective for patients with periodontitis and T2DM. Therefore, more comprehensive studies are needed to elucidate the efficacy of SRP or SRP combined with adjuvant therapy in improving periodontal and glycemic outcomes in this population. This may help identify more optimal treatment modalities and provide evidence-based guidance for clinical practice.

Therefore, the focus of this Bayesian network analysis is to discuss whether SRP in combination with adjuvant treatment has better periodontal and glycemic control efficacy than no intervention or SRP alone in randomized controlled trials (RCTs) regarding patients with periodontitis and T2DM.

## Methods and materials

### Literature search

A search of the Cochrane, PubMed, Embase, and Web of Science databases was conducted for RCTs in patients with periodontitis and T2DM using a combination of MeSH and free-text words was performed as of February 29, 2024. A detailed search strategy is provided in Supplementary Material 1.

### Eligibility criteria

The inclusion criteria for the meta-analysis were defined based on the PICOS guidelines:

- Participants: Adult patients (aged ≥30 years) with no gender, age and career predilection diagnosed as periodontitis and T2DM.
- Intervention: Comparing SRP with no treatment, or comparing SRP with SRP plus adjuvant treatment, or comparing SRP plus adjuvant therapy with different adjuvant therapies.
- Outcome measures: probing depth (PD), clinical attachment level (CAL), bleeding on probing (BOP) [19], HbA1c%, fasting blood sugar (FBS).
- Study design: RCT
- Follow up: At least 3 months

### Exclusion criteria

- Pregnancy and lactation
- Current smoking and smoking within the past 5 years
- Studies including subjects who had systemic conditions (except T2DM) and major complications of T2DM
- Periodontal treatment and antibiotic use within the previous 3 months, Periodontal support therapy within 3 months
- Sample sizes of each group less than 10.

### Study selection and extraction

Two authors (Xuejie Xieand Li Tang) rigorously screened the literature based on predetermined inclusion and exclusion criteria. Any disagreements were resolved through discussion or by consulting a third reviewer (Yiming Li) to reach a consensus. Information extracted from the included studies encompassed the first author, year of publication, country, sample size, sex, mean age, interventions, and outcome measures.

### Quality assessment

The risk of bias assessment was conducted following the most recent recommendations of the Cochrane Handbook for Systematic Reviews of Interventions with ROB 2.0 [20]. This tool includes five main components: bias arising from the randomization, bias due to deviations from intended interventions, bias due to missing outcome data, bias due to measurement of the outcome, and bias due to selective reporting of results. The quality of studies was rated as ‘low risk of bias’, ‘some concerns’, or ‘high risk of bias’. The results were checked by two researchers, and disagreements were resolved through discussion or consultation with a third reviewer to reach a consensus.

### Data analysis

Bayesian network meta-analysis was performed using a prior vague random-effects model with the R 4.2.3 (R Foundation for Statistical Computing). The Markov chain Monte Carlo method [20] was also employed, to obtain the best pooled estimate and probabilities of each treatment regimen. Continuous outcomes were presented as the posterior mean difference (MD) with 95% credible interval (CrI). The surface under the cumulative ranking curve (SUCRA) was calculated to estimate the probability of each intervention being the most effective. Network plots and funnel plots were generated using Stata v15.0. In the network plots, each node represented a medication, and the edges represented the existing comparisons. The size of each node was proportional to the number of patients included. The ggplot2 package was used to draw cumulative probability plots.

## Results

### Study selection

**Figure 1:**
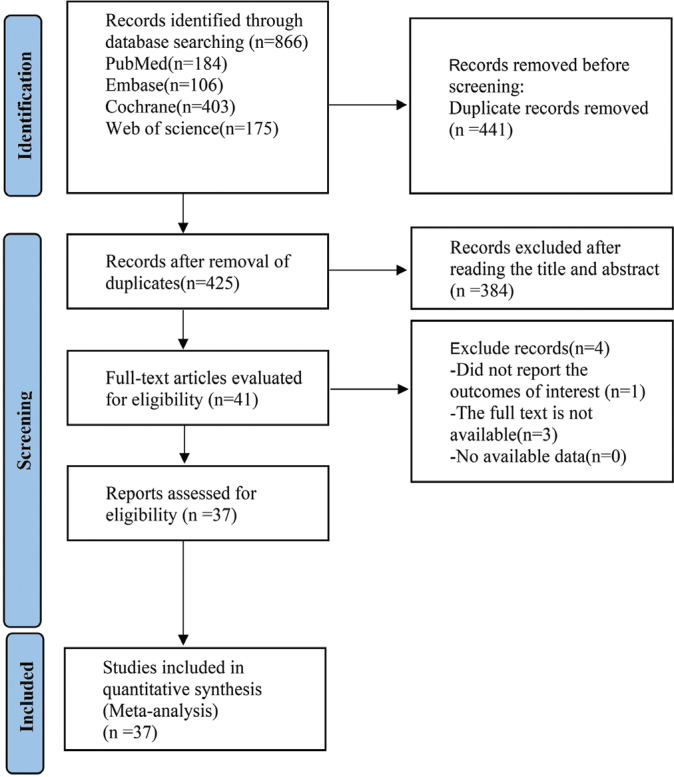
Flow chart of literature screening.

The literature search identified 866 potentially eligible articles. After removing 441 duplicates, 384 articles were excluded based on titles and abstracts. An additional 4 articles were excluded following full-text review. Ultimately, 37 articles were included in this analysis (Figure 1).

### Characteristics of included studies

This meta-analysis evaluated data from 37 eligible studies, including 34 studies [12, 14, 21–52] extracted from published literature and 3 studies sourced from international clinical trial registries (PACTR202111744581541, WHO ICTRP; NCT03374176 and NCT04830969, ClinicalTrials.gov).The analysis encompassed a total of 1,989 patients diagnosed with both periodontitis and T2DM. The evaluated adjunctive treatments included SZ, ZLN, AMX, MTZ, AZM,Doxy, minocycline, DL, ERL, LLLT, aPDT, GSE, melatonin, synbiotic, omega-3, and ginger. Detailed characteristics of the included studies and corresponding references are presented in Table 1.

**Table 1:** Basic characteristics of included studies.

First author	Year	Country	Sample size	Gender (M/F)	Mean age	Intervention	Outcome
Acharya	2021	India	SRP: 24 SRP_GSE:24	Na	30–60	SRP GSE:Systemic 200 mg/day for 8 weeks.	PD;CAL; HbA1c%;FBS
Bazyar	2019	Iran	SRP:22 SRP_melatonin:22	14/30	SRP:51.45 SRP_melatonin:53.72	SRP melatonin:Systemic 3 mg/day for 8 weeks.	PD;CAL
Bazyar	2020	Iran	SRP:24 SRP_Synbiotic:23	14/33	SRP:50.1 SRP_Synbiotic:48.6	SRP Synbiotic:Systemic 500 mg/day and 100 mg fructooligosaccharide for 8 weeks.	PD;CAL
Castro	2016	Brazil	SRP:20 SRP_aPDT:20	10/30	51.60	SRP aPDT:0.005% methylene blue, with wavelength of 660 nm	PD; CAL;
Castro	2020	Brazil	SRP:25 SRP_omega3 a:25 SRP_omega3:25	44/31	SRP:54.9 SRP_omega3 a:55.6 SRP_omega3:55.6	SRP SRP_omega3a: Systemic 900 mg/day ω-3 PUFA and 100 mg/day ASA for 2 months after SRP. SRP_omega3: Systemic 900 mg/day ω-3 PUFA and 100 mg/day ASA for 2 months before SRP.	BOP; CAL; PD
Chandra	2019	India	SRP:18 SRP_DL:18	18/18	SRP:50.6 SRP_DL:48.05	SRP DL(808 nm,1.5W–1.8W)	PD;CAL; HbA1c%
Cruz	2021	Brazil	SRP:10 SRP_AMX_MTZ:15	7/18	SRP:60.5 SRP_AMX_MTZ:60.5	SRP AMX_MTZ:MTZ 400 mg tid and AMX 500 mg tid for 14 days	PD;CAL; BOP
da Silva	2022	Brazil	SRP:11 SRP_ILIB:10	5/16	45–77	SRP ILIB:wavelength: 660 mm; energy density: 6.428 J/cm2	PD;BOP; CAL;HbA1c%; FBS
Eltas	2019	Turkey	SRP:19 SRP_DL:18	17/20	SRP:51.85 SRP_DL:49.7	SRP DL: 810 nm,1W, 400 μm fiber optic tip	CAL;BOP; PD;HbA1c%
Engebretson	2013	New York	OHI:233 SRP:240	277/237	OHI:57.9 SRP:56.7	OHI SRP	PD;BOP; CAL
Feng	2023	China	SRP:22(44 teeth) SRP_ERL:22(44 teeth)	11/11	25–60	SRP ERL:standard tip (72,559), medium- short pulse width (100 μs), energy level of 100 mJ, pulse repetition rate of 10 Hz, average power of 1.0W, water 8, and air 4.	BOP; CAL;PD
Kamatham	2022	India	SRP:14 SRP_LLLT:16	16/14	SRP:46.43 SRP_LLLT:45	SRP LLLT( 630–670 nm,4.0W)	BOP; CAL;PD
Katagiri	2009	Japan	OHI:17 SRP_minocycline :32	27/22	OHI:59.0 SRP_minocycline_SPT:60.3	OHI SRP Minocycline: 10 mg for LDD	PD;BOP
Komatsu	2022	Japan	OHI:22 SRP_AZM:24	17/29	OHI:63.5 SRP_AZM:66.7	OHI SRP AZM:Systemic 2,000 mg/day three days before SRP	PD;BOP; HbA1c%
Kondzielnik	2009	Poland	SRP:19 SRP_Doxy:19	17/21	SRP:55.3 SRP_Doxy:57.7	SRP Doxy:Systemic doxy 20 mg bid for 3 months	PD; CAL;BOP
Kumar	2015	India	OHI:15 SRP_Doxy:15	17/13	>30	OHI SRP Doxy:Systemic doxy 100 mg/day for 14 days	CAL;PD; HbA1c%;FBS
Lee	2020	Korea	OHI:20 SRP:20 SRPAT:20	30/30	OHI:74.15 SRP:71.15 SRPAT:72.45	OHI SRP SRPAT:SRP with Additional toothbrushing (Watanabe method) was performed once a week from the first visit through the fifth visit.	PD;BOP;
Macedo	2014	Brazil	SRP_Doxy:15 SRP_Doxy_aPDT:15	16/29	48.73	SRP Doxy:100 mg/day for 2 weeks after an initial dose of 200 mg aPDT:diode laser (660 nm) with a phenothiazine chloride photosensitizer (10 mg/mL)	PD;BOP; CAL;HbA1c%
Miranda	2014	Brazil	SRP:27 SRP_AMX_MTZ:29	30/26	SRP:53.7 SRP_AMX_MTZ:54.0	SRP MTZ:400 mg tid for 14 days. AMX:500 mg tid for 14 days.	BOP;PD; CAL;HbA1c%; FBS
Arieh	2017	Mexico	SRP_Clindamycin:21 SRP_AMX_MTZ:21	18/24	SRP_Clindamycin:52.0 SRP_AMX_MTZ:52.5	Clindamycin:Systemic Clindamycin 300 mg tid for 7 days. AMX:systemic AMX 500 mg tid for 7 days. MTZ:systemic MTZ 250 mg tid for 7 days.	PD;BOP;
Robert	2021	New York	SRP:23 SRP_SPT:21	33/11	SRP:66 SRP_SPT:60.9	SRP SPT:SRP plus CHX gluconate mouthrinse (15 mL bid for 3 months) and a rubber interdental bristle cleaner (bid for all 12 months of the study. )	PD; CAL;BOP;
O’Connell	2008	Brazil	SRP:15 SRP_Doxy:15	14/16	SRP:53.5 SRP_Doxy:52.3	SRP Doxy:systemic doxy 100 mg/day for 14 days	PD;CAL;BOP;HbA1c%;FBS
Özberk	2020	Turkey	SRP:22 SRP_DL:22	10/12	45.32	SRP DL (980-nm,0.4W)	CAL;PD
Babatope	2020	Nigeria	OHI:27 OHI_SRP:27	18/36	OHI:63.37 SRP:64.41	OHI SRP	PD;CAL;BOP;HbA1c%;FBS
Priyanka	2015	India	SRP:29 SRP_SZ:28	36/28	SRP:42.2 SRP_SZ:40.3	SRP SZ:0.1 mL SZ gel (3%) for LDD	PD;CAL
Promsudth	2005	Thailand	OHI:25 SRP_Doxy:27	19/33	OHI:61.64 SRP_Doxy:61.11	OHI SRP Doxy:systemic doxy 100 mg/day for 2 weeks	PD;CAL; BOP;HbA1c%; FBS
Pulivarthi	2022	India	SRP:14 SRP_LLLT:16	16/14	SRP:46.43 SRP_LLLT:45	SRP LLLT:630–670 nm,0.8W, apical-coronal direction for 15s per site including both lingual, buccal and interproximal sites.	PD;CAL;
Raj	2023	India	SRP:30 SRP_ZLN:30	13/14	43.8	SRP ZLN: After 4-weeks, the sites with persistent pocket of ≥5 mm and intrabony defect of≥3 mm were selected for LDD application. (0.05% ZLN gel 20 μL)	PD;CAL;
Raman	2014	Malaysia	OHI:17 SRP:15	20/12	OHI:54.6 SRP:57.7	OHI SRP:SRP and 0.12% CHX 15 mL tid for 14 day	PD;CAL; HbA1c%
Ramaprabha	2023	India	SRP:21 SRP_vitaminD3:25	46/0	35–60	SRP vitaminD3:60,000 IU once a week for 8 weeks	PD;CAL
Ramos	2016	Brazil	SRP_aPDT:15 SRP_Doxy:15	14/16	SRP_aPDT:48.9 SRP_Doxy:49.3	SRP aPDT:10 mg/mL phenothiazine chloride irradiation by a red laser for 10s at each site (70 mW of power, total energy of 2.79 J/cm² per site) Doxy: 100 mg/day for 14 days with a first dose of 200 mg.	PD;BOP; HbA1c%; CAL
Shashikumar	2022	India	SRP:24 SRP_noni:24	27/21	SRP:47 SRP_noni:48	SRP noni: rinse with 10 mL of noni twice daily.	BOP; CAL;PD
Singh	2008	India	OHI:15 SRP:15 SRP_Doxy:15	Na	>30	OHI SRP Doxy:100 mg/day for 14 days.	PD;CAL; HbA1c%;FBS
Soi	2021	India	SRP:18 SRP_DL:19	30–65	SRP:51.67 SRP_DL:51.58	SRP DL:940 nm ,0.8W	PD;CAL; HbA1c%
Sufaru	2022	Switzerland	SRP:25 SRP_aPDT:24	27/22	SRP:55.24 SRP_aPDT:55.58	SRP aPDT:5 mg/mL indocyanine green irradiation by an 810 nm diode laser	PD;CAL; BOP;HbA1c%; FBS
Toyama	2014	Brazil	FMD:10 SRP:10	9/11	44.5	FMD: disinfection of the full oral cavity within 24 h. SRP	BOP;PD; CAL
Zare	2019	Iran	SRP:21 SRP_ginger:21	19/23	SRP:51.62 SRP_ginger:52.81	SRP ginger:2 g/d (4 tablets of 500 mg as two tablets before lunch and dinner) ginger	PD;CAL

M/F: Male/Female; SRP: Scaling and root planing; GSE: grape seed formulation; aPDT: Antimicrobial photodynamic therapy; ILIB: Modified intravascular laser irradiation of blood; AMX: Amoxicillin; MTZ: Metronidazole; DL: Diode laser; AZM: Azithromycin; ERL: Er-YAG laser; Na: not available; LLLT: Low-level laser therapy; Doxy: Doxycycline; SZ: Satranidazole; CHX: Chlorhexidine; ZLN: 0.05% zoledronate gel; OHI: Oral hygiene instructions; noni: Morindacitrifolia L. mouthwash; FMD: One-stage full-mouth disinfection; ERP: Enhanced root planing; LDD: local drug delivery; PD: Probing Depth; CAL: Clinical Attachment Level; BOP: Bleeding on probing; HbA1c%: Glycated Hemoglobin; FBS: Fasting Blood Sugar.

### Risk of bias in included studies

**Figure 2:**
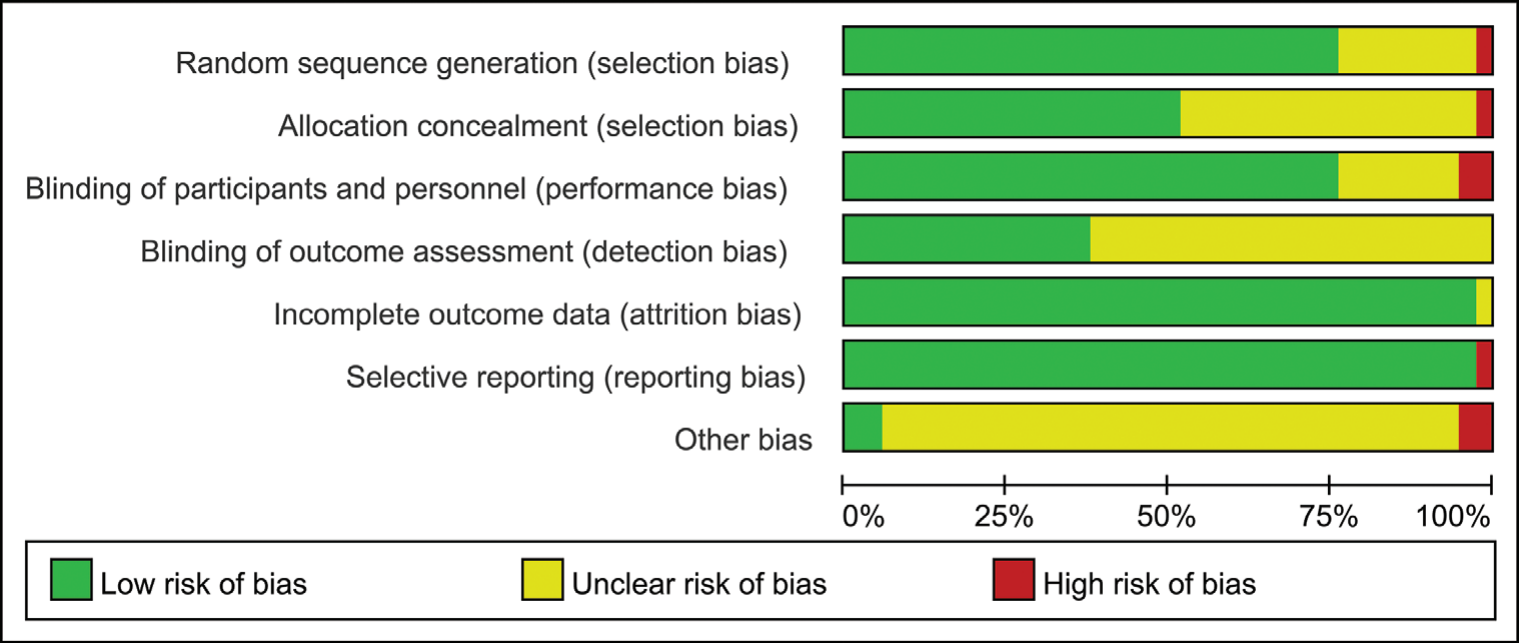
Risk of bias graph.

The included studies were assessed using the Cochrane risk of bias tool. The most common issues were allocation concealment and blinding of participants and personnel. Specifically, 62.1% of the studies did not use blinding for outcome assessors, 48.6% of the studies had unclear or high risk regarding allocation concealment, and 24.3% of the studies did not report detailed information on the randomization method. Additionally, one study was evaluated as having a high risk of selective reporting bias. The risk of bias summary is depicted in Figure 2.

### Efficacy of different treatments for the reduction of PD

**Figure 3:**
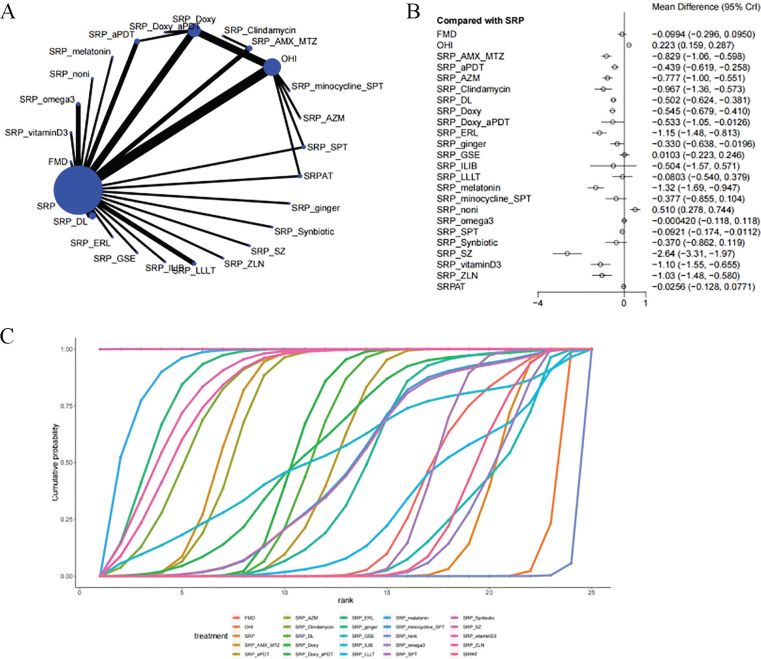
Meta-analysis of probing depth (A: Network plot, B: forest plot, C: area under the cumulative probability curve). SRP: Scaling and root planing; GSE: grape seed formulation; aPDT: Antimicrobial photodynamic therapy; ILIB: Modified intravascular laser irradiation of blood; AMX: Amoxicillin; MTZ: Metronidazole; DL: Diode laser; AZM: Azithromycin; ERL: Er-YAG laser; LLLT: Low-level laser therapy; Doxy: Doxycycline; SZ: Satranidazole; ZLN: 0.05% zoledronate gel; OHI: Oral hygiene instructions; noni: Morindacitrifolia L. mouthwash; FMD: One-stage full-mouth disinfection.

Thirty-seven studies reported PD as an outcome measure for evaluating the efficacy of periodontal treatment. As shown in the network plot (Figure 3A), a closed loop was observed, and thus a local inconsistency test was performed. The results (Supplementary Material 2, Figure S1) indicated no significant differences in direct comparisons, indirect comparisons, and network comparisons. As shown in Figure 3B, compared to SRP alone, superior improvements in PD were noted with SRP+SZ (–2.64 [–3.31, –1.97]), SRP+ERL (–1.15 [–1.48, –0.813]), SRP+melatonin (–1.32 [–1.69, –0.947]). SRP+SZ improved PD more significantly than other treatments (Supplementary Material 2, Table S1). The SUCRA showed the local adjunctive treatment with SZ (100%) was most likely to be the most effective measure for improving PD, followed by systemic treatment with melatonin (92.3%) and local adjunctive treatment with ERL (87.4%), whereas OHI ranked last (5.2%) (Table 2).

**Table 2:** Comprehensive ranking of SUCRA.

Treatment	Probing depth (%)	Clinical attachment level (%)	Bleeding on probing (%)	Glycated hemoglobin (%)	Fasting blood sugar (mg/dL)
OHI	5.2	3.8	5.5	13.9	32.7
SRP	17.7	16.8	23.8	34.5	42.2
SRP_DL	55.1	69.3	67.1	70.9	94.9
SRP_LLLT	NR	19.8	20.0	NR	NR
SRP_aPDT	50.6	59.1	88.0	40.8	NR
SRP_ERL	87.4	70.9	38.1	NR	NR
SRP_SZ	100.0	99.8	NR	NR	NR
SRP_AZM	71.5	NR	99.8	49.8	NR
SRP_minocycline	46.8	NR	60.0	NR	NR
SRP_Doxy	58.5	48.1	83.9	74.9	66.6
SRP_AMX_MTZ	73.7	58.3	61.5	8.7	20.8
SRP_Clindamycin	80.3	NR	79.8	NR	NR
SRP_Doxy_aPDT	56.5	53.2	73.7	95.4	NR
SRP_GSE	18.8	11.0	NR	54.4	40.9
SRP_noni	0.3	24.1	29.7	NR	NR
SRP_ omega3	18.6	32.4	47.8	NR	NR
SRP_ILIB	52.5	51.6	1.1	56.7	52.0
SRP_melatonin	92.3	90.5	NR	NR	NR
SRP_Synbiotic	46.3	51.0	NR	NR	NR
SRP_vitaminD3	85.0	88.9	NR	NR	NR
SRP_ZLN	82.3	84.2	NR	NR	NR

NR: not report; SUCRA: surface under the cumulative ranking curve; OHI: Oral hygiene instructions; SRP: Scaling and root planing; DL: Diode laser; LLLT: Low-level laser therapy; aPDT: Antimicrobial photodynamic therapy; ERL: Er-YAG laser; SZ: Satranidazole; AZM: Azithromycin; AMX: Amoxicillin; MTZ: Metronidazole; GSE: grape seed formulation; ILIB: Modified intravascular laser irradiation of blood; ZLN: 0.05% zoledronate gel.

### Efficacy of different treatments for the reduction of CAL

CAL is defined as the sum of PD (pocket depth) and GR (gingival recession). Thirty-three studies reported CAL as an outcome measure for evaluating the efficacy of periodontal treatment. As illustrated in the network plot (Supplementary Material 2, Figure S3), a closed loop was observed, and a local inconsistency test was conducted. The results (Supplementary Material 2, Figure S4) indicated no significant differences in direct comparisons, indirect comparisons, and network comparisons. As shown in Supplementary Material 2, Figure S3B, compared to SRP alone, superior improvements in CAL were noted with SRP+SZ (–2.28 [–2.90, –1.66]), SRP+melatonin (–1.23 [–1.53, –0.927]), SRP+vitamin D3 (–1.18 [–1.56, –0.808]), SRP+SZ was superior to other adjunctive treatment (as shown in Supplementary Material 2, Table S1). The SUCRA unveiled that local adjunctive treatment with SZ (99.8%) had the highest probability of being the most effective approach for alleviating CAL, followed by systemic treatment with melatonin (90.5%), systemic adjunctive treatment with vitamin D3 (88.9%), whereas OHI ranked last (3.8%) (Table 2).

### Efficacy of different treatments for the reduction of BOP

BOP is defined as the percentage of bleeding sites out of six probing sites per tooth in participants, where 0 indicates no gingival bleeding and 1 indicates the presence of gingival bleeding. Thirty-two studies used BOP as an outcome measure to evaluate periodontal treatment efficacy. As shown in the network plot (Supplementary Material 2, Figure S6A), a closed loop was observed, and thus a local inconsistency test was executed. The results (Supplementary Material 2, Figure S7) suggested no significant differences in direct comparisons, indirect comparisons, and network comparisons between SRP and OHI, SRP+SPT and OHI, SRP+aPDT and SRP, SRP+Doxy and SRP, SRP+SPT and SRP, as well as SRP+Doxy and SRP+aPDT. However, differences were noted in direct, indirect, and network comparisons between SRP+Doxy and OHI. As shown in Supplementary Material 2, Figure S6B, compared to SRP alone, superior improvements in BOP were noted with SRP+AZM (–31.3 [–39.1, –23.5]), SRP+aPDT (–17.6 [–23.3, –11.9]), and SRP+Doxy (–16.1 [–20.9, –11.3]). The SUCRA showed that systemic adjunctive treatment with AZM (99.8%) was most likely to be the most efficacious strategy for improving BOP, followed by local adjunctive treatment with aPDT (88.0%) and systemic adjunctive treatment with Doxy (83.9%) (Table 2).

### Efficacy of different treatments for the reduction of HbA1c%

**HbA1c%** reflects average blood glucose levels over the preceding 3 months. Among the studies included in this research, 16 adopted HbA1c% as an indicator to evaluate glycemic control. As shown in Supplementary Material 2, Figure S9A, a closed loop was formed. After conducting a local inconsistency test, the results (Supplementary Material 2, Figure S10) indicated no significant differences in direct, indirect, or network comparisons between SRP and SRP + adjunctive therapies. As shown in Supplementary Material 2, Figure S9B, compared to SRP alone, SRP+Doxy+aPDT (–1.14 [–1.99, –0.29]) and SRP+DL (–0.27 [–0.46, –0.07]) demonstrated superior effectiveness, with SRP+Doxy+aPDT had the best effect over other adjunctive therapies, followed by SRP+DL. No statistically significant differences were observed between other adjunctive therapies and SRP alone. SUCRA analysis showed that SRP+Doxy+aPDT (95.4%) had the best effect on HbA1c% control, followed by SRP+DL (70.9%) (Table 2).

### Efficacy of different treatments for the reduction of FBS

Nine studies used FBS as an indicator to evaluate glycemic control. As shown in Supplementary Material 2, Figure S12A, a closed loop was formed. The results (Supplementary Material 2, Figure S13) indicated no significant differences in direct, indirect, or network comparisons between SRP and SRP + adjunctive treatments. As shown in Supplementary Material 2, Figure S12B, compared to SRP alone, SRP+DL (–38.6 [–61.8, –15.6]) demonstrated superior effectiveness, with SRP+DL being better than other adjunctive treatments. No statistically significant differences were observed between other adjunctive treatments and SRP alone.

SUCRA analysis showed that SRP+DL (94.9%) was the most effective for FBS control (Table 2).

### Publication bias assessment

The possibility of publication bias regarding the outcome measures PD, CAL, and BOP was evaluated using funnel plots. The results suggested that there was a high possibility of publication bias in PD, CAL, BOP, HbA1c%, and FBS (Supplementary Material 2, Figures S2, S5, S8, S11, S14).

## Discussion

This meta-analysis includes 37 RCTs involving 1989 patients, comparing the effects of different treatments on reducing PD, CAL, BOP, HbA1c%, and FBS. Evidence-based data on the impact of periodontal treatment in controlling periodontal inflammation in T2DM patients are limited. While some studies have compared SRP with no treatment or SRP combined with adjunctive therapies [53, 54] there remains a paucity of data specifically comparing different adjunctive treatments. Our study addresses this gap by including all periodontal treatments that met our inclusion criteria, providing a comprehensive evaluation of their effectiveness.

Antibiotics are commonly used in periodontal treatment to reduce bacterial load, disrupt bacterial protein synthesis and quorum sensing, and inhibit the maturation of biofilms formed by pathogenic microorganisms. This helps control biofilm formation and bacterial colonization within periodontal pockets. For patients with periodontitis and T2DM, the immune system is often compromised, leading to delayed healing. Antibiotics can also modulate the host immune response by reducing the release of inflammatory mediators such as tumor necrosis factor-α (TNF-α) and interleukin-1β (IL-1β). This alleviates inflammation in periodontal tissues, promotes healing, and improves PD, CAL, and BOP [55, 56]. This meta-analysis included comparisons of SRP vs. SRP+AMX+MTZ, SRP vs. SRP+Doxy, SRP vs. SRP+SZ, SRP vs. SRP+minocycline, SRP vs. SRP+clindamycin, and SRP vs. SRP+AZM. Among these, SRP+SZ showed the greatest improvement in PD (–2.64 [–3.31, -1.97]) and CAL (–2.28 [–2.90, –1.66]), while SRP+AZM demonstrated better effectiveness in reducing BOP (–31.3 [–39.1, –23.5]). However, the high heterogeneity of the included studies may affect the interpretation of the results. In addition, the efficacy of different antibiotics varies, and they may cause side effects such as gastrointestinal discomfort and allergic reactions, potentially affecting patient compliance. The choice of antibiotics should be based on individual patient conditions and the characteristics of the pathogenic microorganisms.

In recent years, lasers have been widely used in nonsurgical periodontal therapy (NSPT) due to their beneficial effects on wound healing and local inflammation control [10, 57]. Studies by Qadri and Meimandi et al. [58, 59] have reported positive clinical outcomes from using lasers and aPDT as adjunctive therapies for periodontitis, which aligns with the findings of our study. However, Pourabbas and Sgolastra et al. reported contradictory and short-term findings in clinical studies when comparing the adjunctive effects of aPDT to nonsurgical mechanical instrumentation alone [60, 61]. In Ruoyan Cao’s network meta-analysis [62], SRP+DL showed no significant advantage (0.25 [–0.24, 0.78]). However, in the present study, DL may enhance the efficacy of SRP in reducing HbA1c% (–0.27 [–0.46, –0.07]). This study included comparisons such as SRP+aPDT vs. SRP, SRP+DL vs. SRP, SRP+aPDT vs. SRP+Doxy, SRP+Doxy+aPDT vs. SRP+Doxy, SRP+ERL, and SRP+LLLT. Among these: SRP+ERL was relatively effective in improving PD (–1.15 [–1.48, –0.81]) and CAL (–0.70 [–1.12, –0.28]); SRP+aPDT showed better improvement in BOP (–17.6 [–23.3, –11.9]); SRP+Doxy+aPDT was more effective in reducing HbA1c% (–1.14 [–1.99, –0.29]); SRP+DL showed better efficacy in reducing both HbA1c% (–0.27 [–0.46, –0.07]) and FBS (–38.6 [–61.8, –15.6]). However, due to the limited number of RCTs, it is difficult to draw reliable conclusions. The long-term efficacy and safety of laser therapy require further validation. Future studies should focus on designing rigorous RCTs with longer follow-up periods to reduce the risk of bias and provide stronger evidence.

In other included studies, SRP+melatonin showed relatively better efficacy in improving PD (–1.32 [–1.69, –0.95]) and CAL (–1.23 [–1.53, –0.93]) compared to SRP alone. However, no adjunctive formulation demonstrated superior efficacy in improving BOP over SRP alone. Notably, SRP+melatonin was evaluated in only one trial involving 44 patients. Due to the limited number of RCTs, it is challenging to draw reliable conclusions. Therefore, more well-conducted, multicenter trials are needed to provide stronger evidence.

### Limitations

Although this study explored the differences in periodontal efficacy between SRP and various adjunctive treatments, we must note the following limitations. First, the included studies did not analyze PD, CAL, BOP as primary outcome indicators. Second, there was considerable heterogeneity in the treatment methods among the study populations. We need more research to substantiate our viewpoints, but this study also provides a treatment option for patients with periodontitis and T2DM.

## Conclusions

The results of this meta-analysis seem to support that periodontal treatment with SRP+SZ possesses the best efficacy in lowering PD and CAL of periodontitis complicated by T2DM, while SRP+AZM showed the greatest efficacy in reducing BOP. Additionally, SRP+Doxy+aPDT appeared to be more effective in improving HbA1c%, and SRP+DL showed better efficacy in improving FBS. However, the quality of evidence is low or very low, and therefore further studies are needed to confirm the results.

## Supplementary Material

Efficacy of nonsurgical periodontal treatment on patients with periodontitis and type 2 diabetes mellitus: a systematic review and Bayesian network meta-analysis

Efficacy of nonsurgical periodontal treatment on patients with periodontitis and type 2 diabetes mellitus: a systematic review and Bayesian network meta-analysis
