# Supplementary material for: Efficacy of nonsurgical periodontal treatment on patients with periodontitis and type 2 diabetes mellitus: a systematic review and Bayesian network meta-analysis
Source: Acta Odontol Scand. 2025 May 13;84:43344. doi: 10.2340/aos.v84.43344 (PMC12095944; doi:10.2340/aos.v84.43344)
Supplement: Efficacy of nonsurgical periodontal treatment on patients with periodontitis and type 2 diabetes mellitus: a systematic review and Bayesian network meta-analysis [file AOS-84-43344-s1.pdf]

Supplementary material has been published as submitted. It has not been copyedited or typeset by Acta Odontologica Scandinavica.

Cochrane

ID Search Hits

#1 MeSH descriptor: [Periodontal Diseases] explode all trees 8417

#2 (Periodontal Diseases):ti,ab,kw OR (Disease, Periodontal):ti,ab,kw OR (Diseases, Periodontal):ti,ab,kw OR (Periodontal Disease):ti,ab,kw OR (Parodontosis):ti,ab,kw (Word variations have been searched) 5728

#3 (Parodontoses):ti,ab,kw OR (Pyorrhea Alveolaris):ti,ab,kw (Word variations have been searched) 0

#4 #1 or #2 or #3 11675

#5 MeSH descriptor: [Diabetes Mellitus, Type 2] explode all trees 23475

#6 (Diabetes Mellitus, Type 2):ti,ab,kw OR (Diabetes Mellitus, Noninsulin-Dependent):ti,ab,kw OR (Diabetes Mellitus, Ketosis-Resistant):ti,ab,kw OR (Diabetes Mellitus, Ketosis Resistant):ti,ab,kw OR (Ketosis-Resistant Diabetes Mellitus):ti,ab,kw (Word variations have been searched) 51508

#7 (Diabetes Mellitus, Type II):ti,ab,kw OR (NIDDM):ti,ab,kw OR (Diabetes Mellitus, Noninsulin Dependent):ti,ab,kw OR (Diabetes Mellitus, Maturity-Onset):ti,ab,kw OR (Diabetes Mellitus, Maturity Onset):ti,ab,kw (Word variations have been searched) 6677

#8 (Diabetes Mellitus, Non Insulin Dependent):ti,ab,kw OR (Diabetes Mellitus, Non-Insulin-Dependent):ti,ab,kw OR (Non-Insulin-Dependent Diabetes Mellitus):ti,ab,kw OR (Diabetes Mellitus, Stable):ti,ab,kw OR (Stable Diabetes Mellitus):ti,ab,kw (Word variations have been searched) 25043

#9 (Maturity-Onset Diabetes Mellitus):ti,ab,kw OR (Maturity Onset Diabetes Mellitus):ti,ab,kw OR (MODY):ti,ab,kw OR (Diabetes Mellitus, Slow-Onset):ti,ab,kw OR (Diabetes Mellitus, Slow Onset):ti,ab,kw (Word variations have been searched) 315

#10 (Slow-Onset Diabetes Mellitus):ti,ab,kw OR (Type 2 Diabetes Mellitus):ti,ab,kw

OR (Noninsulin-Dependent Diabetes Mellitus):ti,ab,kw OR (Noninsulin Dependent Diabetes Mellitus):ti,ab,kw OR (Maturity-Onset Diabetes):ti,ab,kw (Word variations have been searched) 51588

#11 (Diabetes, Maturity-Onset):ti,ab,kw OR (Maturity Onset Diabetes):ti,ab,kw OR (Type 2 Diabetes):ti,ab,kw OR (Diabetes, Type 2):ti,ab,kw OR (Diabetes Mellitus, Adult-Onset):ti,ab,kw (Word variations have been searched) 60325

#12 (Adult-Onset Diabetes Mellitus):ti,ab,kw OR (Diabetes Mellitus, Adult Onset):ti,ab,kw (Word variations have been searched) 1834

#13 #5 or #6 or #7 or #8 or #9 or #10 or #11 or #12 64748

#14 #4 and #13 403

Embase

No. Query Results Date

#48 #34 AND #43 AND #47 106

#47 #44 OR #45 OR #46 867652

#46 'double-blind':ab,ti 226446

#45 'placebo':ab,ti 368597

#44 'random':ab,ti 439662

#43 #35 OR #36 OR #37 OR #38 OR #39 OR #40 OR #41 OR #42 132851

#42 'pyorrhea alveolaris therapy':ab,ti 0

#41 'parodontoses':ab,ti 8

#40 'parodontosis':ab,ti 243

#39 'periodontal disease':ab,ti 22334

#38 'diseases, periodontal':ab,ti 74

#37 'disease, periodontal':ab,ti 161

#36 'periodontal diseases':ab,ti7421

#35 'periodontal disease'/exp 128309

#34 #1 OR #2 OR #3 OR #4 OR #5 OR #6 OR #7 OR #8 OR #9 OR #10 OR #11 OR #12 OR #13 OR #14 OR #15 OR #16 OR #17 OR #18 OR #19 OR #20 OR #21 OR #22 OR #23 OR #24 OR #25 OR #26 OR #27 OR #28 OR #29 OR #30 OR #31 OR

#32 OR #33 394519

#33 'diabetes mellitus, adult onset':ab,ti3

#32 'adult-onset diabetes mellitus':ab,ti 191

#31 'diabetes mellitus, adult-onset':ab,ti 3

#30 'diabetes, type 2':ab,ti 2909

#29 'type 2 diabetes':ab,ti 256439

#28 'maturity onset diabetes':ab,ti 3094

#27 'diabetes, maturity-onset':ab,ti 57

#26 'maturity-onset diabetes':ab,ti 3094

#25 'noninsulin dependent diabetes mellitus':ab,ti 1060

#24 'noninsulin-dependent diabetes mellitus':ab,ti 1060

#23 'type 2 diabetes mellitus':ab,ti 89234

#22 'slow-onset diabetes mellitus':ab,ti 0

#21 'diabetes mellitus, slow onset':ab,ti 1

#20 'diabetes mellitus, slow-onset':ab,ti 1

#19 'mody':ab,ti 2883

#18 'maturity onset diabetes mellitus':ab,ti 172

#17 'diabetes mellitus, maturity onset':ab,ti 15

#16 'diabetes mellitus, maturity onset':ab,ti 15

#15 'diabetes mellitus, maturity-onset':ab,ti 15

#14 'diabetes mellitus, noninsulin dependent':ab,ti 5

#13 'niddm':ab,ti 8113

#12 'diabetes mellitus, type ii':ab,ti 1417

#11 'stable diabetes mellitus':ab,ti 26

#10 'diabetes mellitus, stable':ab,ti 17

#9 'non-insulin-dependent diabetes mellitus':ab,ti 7880

#8 'diabetes mellitus, non-insulin-dependent':ab,ti 43

#7 'diabetes mellitus, non insulin dependent':ab,ti 43

#6 'ketosis-resistant diabetes mellitus':ab,ti 2

#5 'diabetes mellitus, ketosis resistant':ab,ti 0

#4 'diabetes mellitus, ketosis-resistant':ab,ti 0

#3 'diabetes mellitus, noninsulin-dependent':ab,ti 5

#2 'diabetes mellitus, type 2':ab,ti 6030

#1 'non insulin dependent diabetes mellitus'/exp 345855

Pubmed

Search number Query Sort By Filters Search Details Results Time

8 ((randomized controlled trial[Publication Type] OR randomized[Title/Abstract] OR placebo[Title/Abstract]) AND (("Diabetes Mellitus, Type 2"[Mesh]) OR ((((((((((((((((((((((((((((((((Diabetes Mellitus, Type 2[Title/Abstract]) OR (Diabetes Mellitus, Noninsulin-Dependent[Title/Abstract])) OR (Diabetes Mellitus, Ketosis-Resistant[Title/Abstract])) OR (Diabetes Mellitus, Ketosis Resistant[Title/Abstract])) OR (Ketosis-Resistant Diabetes Mellitus[Title/Abstract])) OR (Diabetes Mellitus, Non Insulin Dependent[Title/Abstract])) OR (Diabetes Mellitus, Non-Insulin-Dependent[Title/Abstract])) OR (Non-Insulin-Dependent Diabetes Mellitus[Title/Abstract])) OR (Diabetes Mellitus, Stable[Title/Abstract])) OR (Stable Diabetes Mellitus[Title/Abstract])) OR (Diabetes Mellitus, Type II[Title/Abstract])) OR (NIDDM[Title/Abstract])) OR (Diabetes Mellitus, Noninsulin Dependent[Title/Abstract])) OR (Diabetes Mellitus, Maturity-Onset[Title/Abstract])) OR (Diabetes Mellitus, Maturity Onset[Title/Abstract])) OR (Maturity-Onset Diabetes Mellitus[Title/Abstract])) OR (Maturity Onset Diabetes Mellitus[Title/Abstract])) OR (MODY[Title/Abstract])) OR (Diabetes Mellitus, Slow-Onset[Title/Abstract])) OR (Diabetes Mellitus, Slow Onset[Title/Abstract])) OR (Slow-Onset Diabetes Mellitus[Title/Abstract])) OR (Type 2 Diabetes Mellitus[Title/Abstract])) OR (Noninsulin-Dependent Diabetes Mellitus[Title/Abstract])) OR (Noninsulin Dependent Diabetes Mellitus[Title/Abstract])) OR (Maturity-Onset Diabetes[Title/Abstract])) OR (Diabetes, Maturity-Onset[Title/Abstract])) OR (Maturity Onset Diabetes[Title/Abstract])) OR (Type 2 Diabetes[Title/Abstract])) OR (Diabetes, Type 2[Title/Abstract])) OR (Diabetes Mellitus, Adult-Onset[Title/Abstract])) OR

(Adult-Onset Diabetes Mellitus[Title/Abstract])) OR (Diabetes Mellitus, Adult Onset[Title/Abstract])))) AND (("Periodontal Diseases"[Mesh]) OR (((((((Periodontal Diseases[Title/Abstract]) OR (Disease, Periodontal[Title/Abstract])) OR (Diseases, Periodontal[Title/Abstract])) OR (Periodontal Disease[Title/Abstract])) OR (Parodontosis[Title/Abstract])) OR (Parodontoses[Title/Abstract])) OR (Pyorrhea Alveolaris[Title/Abstract]))) ("randomized controlled trial"[Publication Type] OR "randomized"[Title/Abstract] OR "placebo"[Title/Abstract]) AND ("diabetes mellitus, type 2"[MeSH Terms] OR ("diabetes mellitus type 2"[Title/Abstract] OR "diabetes mellitus noninsulin dependent"[Title/Abstract] OR (("diabetes mellitus"[MeSH Terms] OR ("Diabetes"[All Fields] AND "Mellitus"[All Fields]) OR "diabetes mellitus"[All Fields]) AND "Ketosis-Resistant"[Title/Abstract]) OR (("diabetes mellitus"[MeSH Terms] OR ("Diabetes"[All Fields] AND "Mellitus"[All Fields]) OR "diabetes mellitus"[All Fields]) AND "Ketosis-Resistant"[Title/Abstract]) OR "ketosis resistant diabetes mellitus"[Title/Abstract] OR "diabetes mellitus non insulin dependent"[Title/Abstract] OR "diabetes mellitus non insulin dependent"[Title/Abstract] OR "non insulin dependent diabetes mellitus"[Title/Abstract] OR "diabetes mellitus stable"[Title/Abstract] OR "stable diabetes mellitus"[Title/Abstract] OR "diabetes mellitus type ii"[Title/Abstract] OR "NIDDM"[Title/Abstract] OR "diabetes mellitus noninsulin dependent"[Title/Abstract] OR "diabetes mellitus maturity onset"[Title/Abstract] OR "diabetes mellitus maturity onset"[Title/Abstract] OR "maturity onset diabetes mellitus"[Title/Abstract] OR "maturity onset diabetes mellitus"[Title/Abstract] OR "MODY"[Title/Abstract] OR "diabetes mellitus slow onset"[Title/Abstract] OR "diabetes mellitus slow onset"[Title/Abstract] OR ("Slow-Onset"[All Fields] AND "diabetes mellitus"[Title/Abstract]) OR "type 2 diabetes mellitus"[Title/Abstract] OR "noninsulin dependent diabetes mellitus"[Title/Abstract] OR "noninsulin dependent diabetes mellitus"[Title/Abstract] OR "maturity onset diabetes"[Title/Abstract] OR "diabetes maturity onset"[Title/Abstract] OR "maturity onset diabetes"[Title/Abstract] OR "type 2 diabetes"[Title/Abstract] OR "diabetes type 2"[Title/Abstract] OR "diabetes mellitus

adult onset"[Title/Abstract] OR "adult onset diabetes mellitus"[Title/Abstract] OR "diabetes mellitus adult onset"[Title/Abstract])) AND ("Periodontal Diseases"[MeSH Terms] OR ("Periodontal Diseases"[Title/Abstract] OR "disease periodontal"[Title/Abstract] OR "diseases periodontal"[Title/Abstract] OR "periodontal disease"[Title/Abstract] OR "Parodontosis"[Title/Abstract] OR "Parodontoses"[Title/Abstract] OR "pyorrhea alveolaris"[Title/Abstract])) 184 5:47:23

7 ("Periodontal Diseases"[Mesh]) OR (((((((Periodontal Diseases[Title/Abstract]) OR (Disease, Periodontal[Title/Abstract])) OR (Diseases, Periodontal[Title/Abstract])) OR (Periodontal Disease[Title/Abstract])) OR (Parodontosis[Title/Abstract])) OR (Parodontoses[Title/Abstract])) OR (Pyorrhea Alveolaris[Title/Abstract]))

"Periodontal Diseases"[MeSH Terms] OR "Periodontal Diseases"[Title/Abstract] OR "disease periodontal"[Title/Abstract] OR "diseases periodontal"[Title/Abstract] OR "periodontal disease"[Title/Abstract] OR "Parodontosis"[Title/Abstract] OR "Parodontoses"[Title/Abstract] OR "pyorrhea alveolaris"[Title/Abstract] 105,336 5:46:45

6 ("Diabetes Mellitus, Type 2"[Mesh]) OR ((((((((((((((((((((((((((((((Diabetes Mellitus, Type 2[Title/Abstract]) OR (Diabetes Mellitus, Noninsulin-Dependent[Title/Abstract])) OR (Diabetes Mellitus, Ketosis-Resistant[Title/Abstract])) OR (Diabetes Mellitus, Ketosis Resistant[Title/Abstract])) OR (Ketosis-Resistant Diabetes Mellitus[Title/Abstract])) OR (Diabetes Mellitus, Non Insulin Dependent[Title/Abstract])) OR (Diabetes Mellitus, Non-Insulin-Dependent[Title/Abstract])) OR (Non-Insulin-Dependent Diabetes Mellitus[Title/Abstract])) OR (Diabetes Mellitus, Stable[Title/Abstract])) OR (Stable Diabetes Mellitus[Title/Abstract])) OR (Diabetes Mellitus, Type II[Title/Abstract])) OR (NIDDM[Title/Abstract])) OR (Diabetes Mellitus, Noninsulin Dependent[Title/Abstract])) OR (Diabetes Mellitus, Maturity-Onset[Title/Abstract])) OR (Diabetes Mellitus, Maturity Onset[Title/Abstract])) OR (Maturity-Onset Diabetes Mellitus[Title/Abstract])) OR (Maturity Onset Diabetes Mellitus[Title/Abstract])) OR (MODY[Title/Abstract])) OR (Diabetes Mellitus, Slow-Onset[Title/Abstract])) OR (Diabetes Mellitus, Slow Onset[Title/Abstract])) OR

(Slow-Onset Diabetes Mellitus[Title/Abstract])) OR (Type 2 Diabetes Mellitus[Title/Abstract])) OR (Noninsulin-Dependent Diabetes Mellitus[Title/Abstract])) OR (Noninsulin Dependent Diabetes Mellitus[Title/Abstract])) OR (Maturity-Onset Diabetes[Title/Abstract])) OR (Diabetes, Maturity-Onset[Title/Abstract])) OR (Maturity Onset Diabetes[Title/Abstract])) OR (Type 2 Diabetes[Title/Abstract])) OR (Diabetes, Type 2[Title/Abstract])) OR (Diabetes Mellitus, Adult-Onset[Title/Abstract])) OR (Adult-Onset Diabetes Mellitus[Title/Abstract])) OR (Diabetes Mellitus, Adult Onset[Title/Abstract])) "diabetes mellitus, type 2"[MeSH Terms] OR ("diabetes mellitus type 2"[Title/Abstract] OR "diabetes mellitus noninsulin dependent"[Title/Abstract] OR (("diabetes mellitus"[MeSH Terms] OR ("Diabetes"[All Fields] AND "Mellitus"[All Fields]) OR "diabetes mellitus"[All Fields]) AND "Ketosis-Resistant"[Title/Abstract]) OR (("diabetes mellitus"[MeSH Terms] OR ("Diabetes"[All Fields] AND "Mellitus"[All Fields]) OR "diabetes mellitus"[All Fields]) AND "Ketosis-Resistant"[Title/Abstract]) OR "ketosis resistant diabetes mellitus"[Title/Abstract] OR "diabetes mellitus non insulin dependent"[Title/Abstract] OR "diabetes mellitus non insulin dependent"[Title/Abstract] OR "non insulin dependent diabetes mellitus"[Title/Abstract] OR "diabetes mellitus stable"[Title/Abstract] OR "stable diabetes mellitus"[Title/Abstract] OR "diabetes mellitus type ii"[Title/Abstract] OR "NIDDM"[Title/Abstract] OR "diabetes mellitus noninsulin dependent"[Title/Abstract] OR "diabetes mellitus maturity onset"[Title/Abstract] OR "diabetes mellitus maturity onset"[Title/Abstract] OR "maturity onset diabetes mellitus"[Title/Abstract] OR "maturity onset diabetes mellitus"[Title/Abstract] OR "MODY"[Title/Abstract] OR "diabetes mellitus slow onset"[Title/Abstract] OR "diabetes mellitus slow onset"[Title/Abstract] OR ("Slow-Onset"[All Fields] AND "diabetes mellitus"[Title/Abstract]) OR "type 2 diabetes mellitus"[Title/Abstract] OR "noninsulin dependent diabetes mellitus"[Title/Abstract] OR "noninsulin dependent diabetes mellitus"[Title/Abstract] OR "maturity onset diabetes"[Title/Abstract] OR "diabetes maturity onset"[Title/Abstract] OR "maturity onset diabetes"[Title/Abstract]

OR "type 2 diabetes"[Title/Abstract] OR "diabetes type 2"[Title/Abstract] OR "diabetes mellitus adult onset"[Title/Abstract] OR "adult onset diabetes mellitus"[Title/Abstract] OR "diabetes mellitus adult onset"[Title/Abstract]) 238,973 5:46:09

5 randomized controlled trial[Publication Type] OR randomized[Title/Abstract] OR placebo[Title/Abstract] "randomized controlled trial"[Publication Type] OR "randomized"[Title/Abstract] OR "placebo"[Title/Abstract] 1,034,350 5:45:36

4 ((((((Periodontal Diseases[Title/Abstract]) OR (Disease, Periodontal[Title/Abstract])) OR (Diseases, Periodontal[Title/Abstract])) OR (Periodontal Disease[Title/Abstract])) OR (Parodontosis[Title/Abstract])) OR (Parodontoses[Title/Abstract])) OR (Pyorrhea Alveolaris[Title/Abstract])

"periodontal diseases"[Title/Abstract] OR "disease periodontal"[Title/Abstract] OR "diseases periodontal"[Title/Abstract] OR "periodontal disease"[Title/Abstract] OR "Parodontosis"[Title/Abstract] OR "Parodontoses"[Title/Abstract] OR "pyorrhea alveolaris"[Title/Abstract]28,460 5:42:40

3 "Periodontal Diseases"[Mesh] Most Recent "Periodontal Diseases"[MeSH Terms] 96,449 5:39:12

2 ((((((((((((((((((((((((((((((Diabetes Mellitus, Type 2[Title/Abstract]) OR (Diabetes Mellitus, Noninsulin-Dependent[Title/Abstract])) OR (Diabetes Mellitus, Ketosis-Resistant[Title/Abstract])) OR (Diabetes Mellitus, Ketosis Resistant[Title/Abstract])) OR (Ketosis-Resistant Diabetes Mellitus[Title/Abstract])) OR (Diabetes Mellitus, Non Insulin Dependent[Title/Abstract])) OR (Diabetes Mellitus, Non-Insulin-Dependent[Title/Abstract])) OR (Non-Insulin-Dependent Diabetes Mellitus[Title/Abstract])) OR (Diabetes Mellitus, Stable[Title/Abstract])) OR (Stable Diabetes Mellitus[Title/Abstract])) OR (Diabetes Mellitus, Type II[Title/Abstract])) OR (NIDDM[Title/Abstract])) OR (Diabetes Mellitus, Noninsulin Dependent[Title/Abstract])) OR (Diabetes Mellitus, Maturity-Onset[Title/Abstract])) OR (Diabetes Mellitus, Maturity Onset[Title/Abstract])) OR (Maturity-Onset Diabetes Mellitus[Title/Abstract])) OR (Maturity Onset Diabetes Mellitus[Title/Abstract])) OR (MODY[Title/Abstract])) OR (Diabetes Mellitus,

Slow-Onset[Title/Abstract])) OR (Diabetes Mellitus, Slow Onset[Title/Abstract])) OR (Slow-Onset Diabetes Mellitus[Title/Abstract])) OR (Type 2 Diabetes Mellitus[Title/Abstract])) OR (Noninsulin-Dependent Diabetes Mellitus[Title/Abstract])) OR (Noninsulin Dependent Diabetes Mellitus[Title/Abstract])) OR (Maturity-Onset Diabetes[Title/Abstract])) OR (Diabetes, Maturity-Onset[Title/Abstract])) OR (Maturity Onset Diabetes[Title/Abstract])) OR (Type 2 Diabetes[Title/Abstract])) OR (Diabetes, Type 2[Title/Abstract])) OR (Diabetes Mellitus, Adult-Onset[Title/Abstract])) OR (Adult-Onset Diabetes Mellitus[Title/Abstract])) OR (Diabetes Mellitus, Adult Onset[Title/Abstract]) "diabetes mellitus type 2"[Title/Abstract] OR "diabetes mellitus noninsulin dependent"[Title/Abstract] OR (("diabetes mellitus"[MeSH Terms] OR ("Diabetes"[All Fields] AND "Mellitus"[All Fields]) OR "diabetes mellitus"[All Fields]) AND "Ketosis-Resistant"[Title/Abstract]) OR (("diabetes mellitus"[MeSH Terms] OR ("Diabetes"[All Fields] AND "Mellitus"[All Fields]) OR "diabetes mellitus"[All Fields]) AND "Ketosis-Resistant"[Title/Abstract]) OR "ketosis resistant diabetes mellitus"[Title/Abstract] OR "diabetes mellitus non insulin dependent"[Title/Abstract] OR "diabetes mellitus non insulin dependent"[Title/Abstract] OR "non insulin dependent diabetes mellitus"[Title/Abstract] OR "diabetes mellitus stable"[Title/Abstract] OR "stable diabetes mellitus"[Title/Abstract] OR "diabetes mellitus type ii"[Title/Abstract] OR "NIDDM"[Title/Abstract] OR "diabetes mellitus noninsulin dependent"[Title/Abstract] OR "diabetes mellitus maturity onset"[Title/Abstract] OR "diabetes mellitus maturity onset"[Title/Abstract] OR "maturity onset diabetes mellitus"[Title/Abstract] OR "maturity onset diabetes mellitus"[Title/Abstract] OR "MODY"[Title/Abstract] OR "diabetes mellitus slow onset"[Title/Abstract] OR "diabetes mellitus slow onset"[Title/Abstract] OR ("Slow-Onset"[All Fields] AND "diabetes mellitus"[Title/Abstract]) OR "type 2 diabetes mellitus"[Title/Abstract] OR "noninsulin dependent diabetes mellitus"[Title/Abstract] OR "noninsulin dependent diabetes mellitus"[Title/Abstract] OR "maturity onset diabetes"[Title/Abstract] OR "diabetes maturity onset"[Title/Abstract] OR "maturity onset diabetes"[Title/Abstract]

OR "type 2 diabetes"[Title/Abstract] OR "diabetes type 2"[Title/Abstract] OR "diabetes mellitus adult onset"[Title/Abstract] OR "adult onset diabetes mellitus"[Title/Abstract] OR "diabetes mellitus adult onset"[Title/Abstract] 187,277 5:35:17

1 "Diabetes Mellitus, Type 2"[Mesh] "diabetes mellitus, type 2"[MeSH Terms] 174,425 5:21:34

Web of science

#	search strategy	search results
1	TS=(Diabetes Mellitus, Type 2) OR TS=(Diabetes Mellitus, Noninsulin-Dependent) OR TS=(Diabetes Mellitus, Ketosis-Resistant) OR TS=(Diabetes Mellitus, Ketosis Resistant) OR TS=(Ketosis-Resistant Diabetes Mellitus) OR TS=(Diabetes Mellitus, Non Insulin Dependent) OR TS=(Diabetes Mellitus, Non-Insulin-Dependent) OR TS=(Non-Insulin-Dependent Diabetes Mellitus) OR TS=(Diabetes Mellitus, Stable) OR TS=(Stable Diabetes Mellitus) OR TS=(Diabetes Mellitus, Type II) OR TS=(NIDDM) OR TS=(Diabetes Mellitus, Noninsulin Dependent) OR TS=(Diabetes Mellitus, Maturity-Onset) OR TS=(Diabetes Mellitus, Maturity Onset) OR TS=(Maturity-Onset Diabetes Mellitus) OR TS=(Maturity Onset Diabetes Mellitus) OR TS=(MODY) OR TS=(Diabetes Mellitus, Slow-Onset) OR TS=(Diabetes Mellitus, Slow Onset) OR TS=(Slow-Onset Diabetes Mellitus) OR TS=(Type 2 Diabetes Mellitus) OR TS=(Noninsulin-Dependent Diabetes Mellitus) OR TS=(Noninsulin Dependent Diabetes Mellitus) OR TS=(Maturity-Onset Diabetes) OR TS=(Diabetes, Maturity-Onset) OR TS=(Maturity Onset Diabetes) OR TS=(Type 2 Diabetes) OR TS=(Diabetes, Type 2) OR TS=(Diabetes Mellitus, Adult-Onset) OR TS=(Adult-Onset Diabetes Mellitus) OR TS=(Diabetes Mellitus, Adult Onset	293713

	)	
2	TS=(Periodontal Diseases<br>) OR (TS=Disease, Periodontal) OR TS=(Diseases, Periodontal<br>) OR TS=(Periodontal Disease<br>) OR TS=(Parodontosis<br>) OR TS=(Parodontoses<br>) OR TS=(Pyorrhea Alveolaris<br>)	38961
3	TS=(Random<br>) OR TS=(Placebo<br>) OR TS=(double-blind<br>) OR TS=(randomized controlled trial<br>) OR TS=(Randomized<br>) OR TS=(placebo<br>)	2261138
4	#3 AND #2 AND #1	175
