# Supplementary material for: Efficacy of nonsurgical periodontal treatment on patients with periodontitis and type 2 diabetes mellitus: a systematic review and Bayesian network meta-analysis
Source: Acta Odontol Scand. 2025 May 13;84:43344. doi: 10.2340/aos.v84.43344 (PMC12095944; doi:10.2340/aos.v84.43344)
Supplement: Efficacy of nonsurgical periodontal treatment on patients with periodontitis and type 2 diabetes mellitus: a systematic review and Bayesian network meta-analysis [file AOS-84-43344-s2.pdf]

Supplementary material has been published as submitted. It has not been copyedited or typeset by Acta Odontologica Scandinavica.

Figure S1: Local inconsistency test for PD

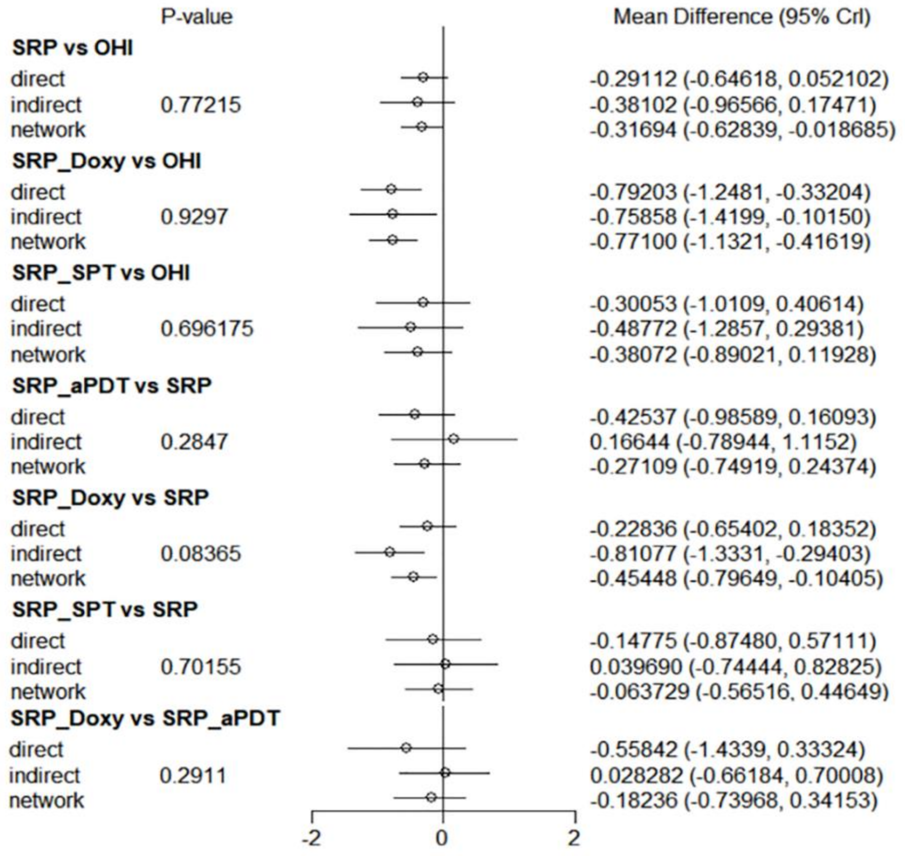

Figure S2: Funnel plot for PD

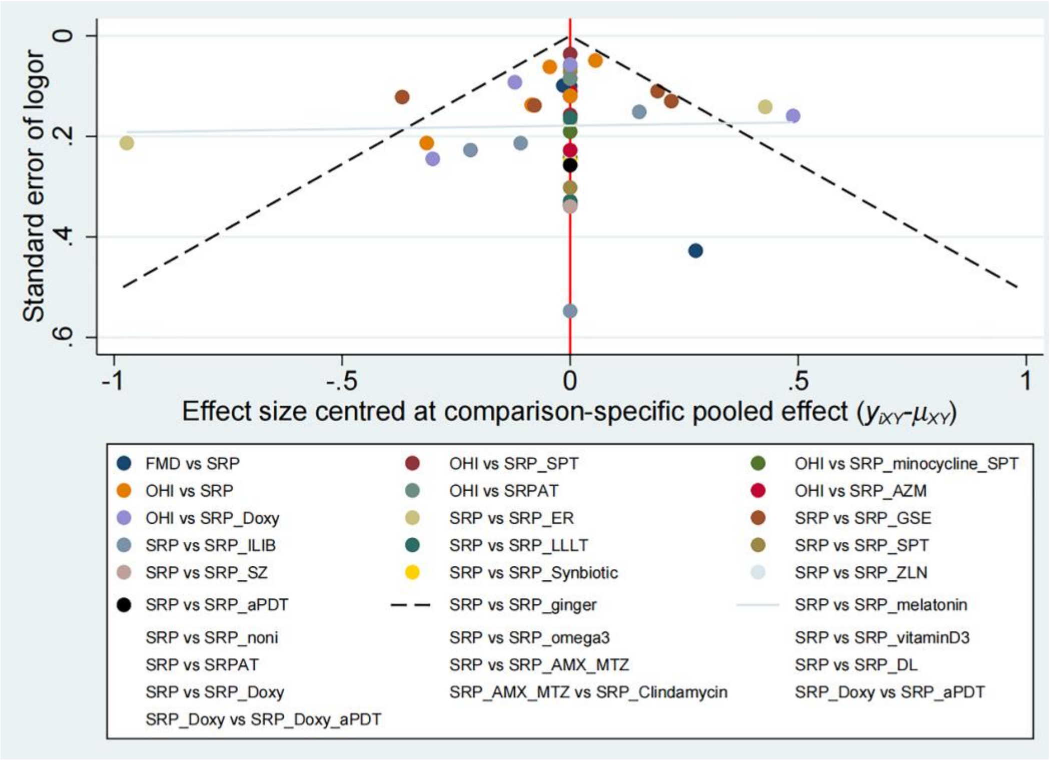

Figure S3: Meta-analysis of CAL

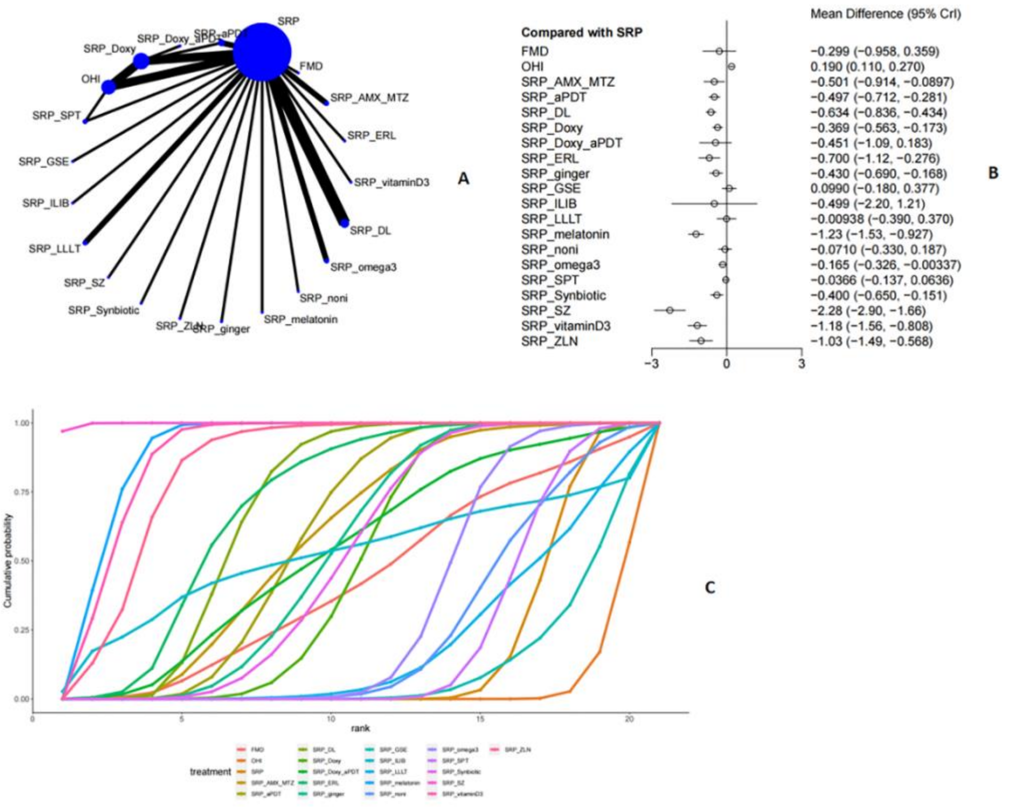

Figure S4: Local inconsistency test for CAL

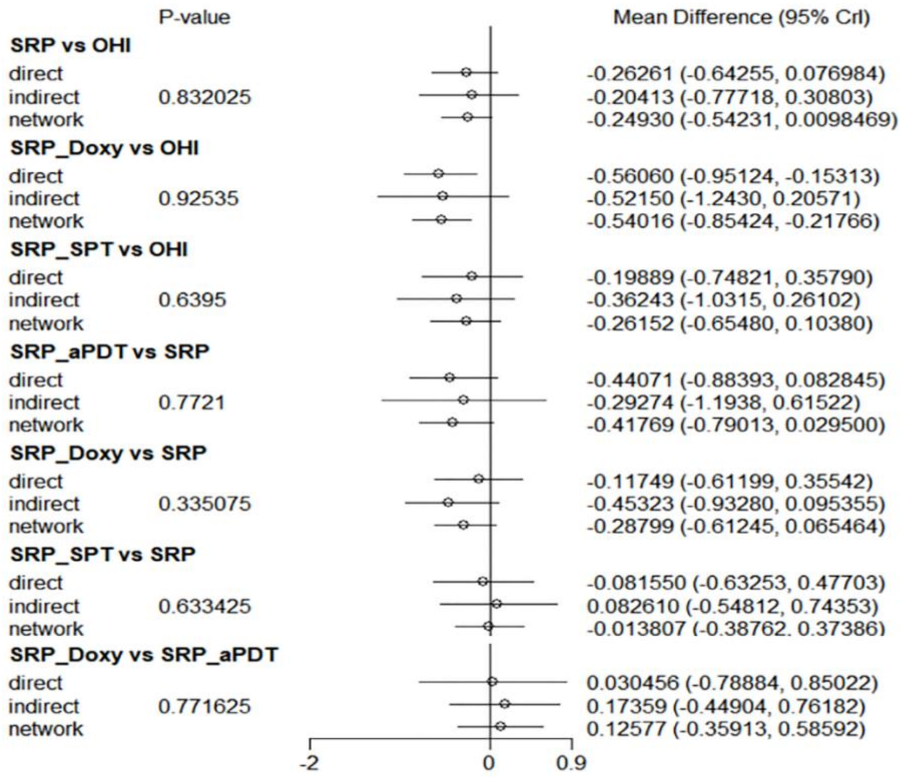

Figure S5: Funnel plot for CAL

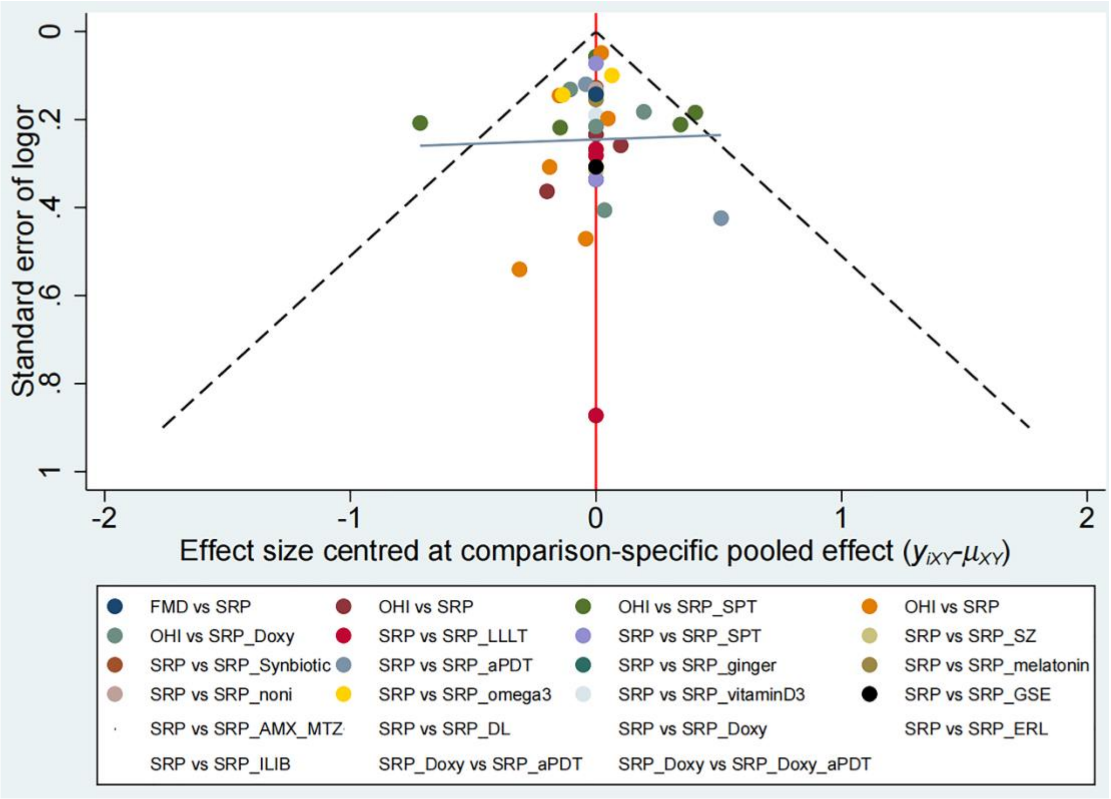

Figure S6: Meta-analysis of BOP

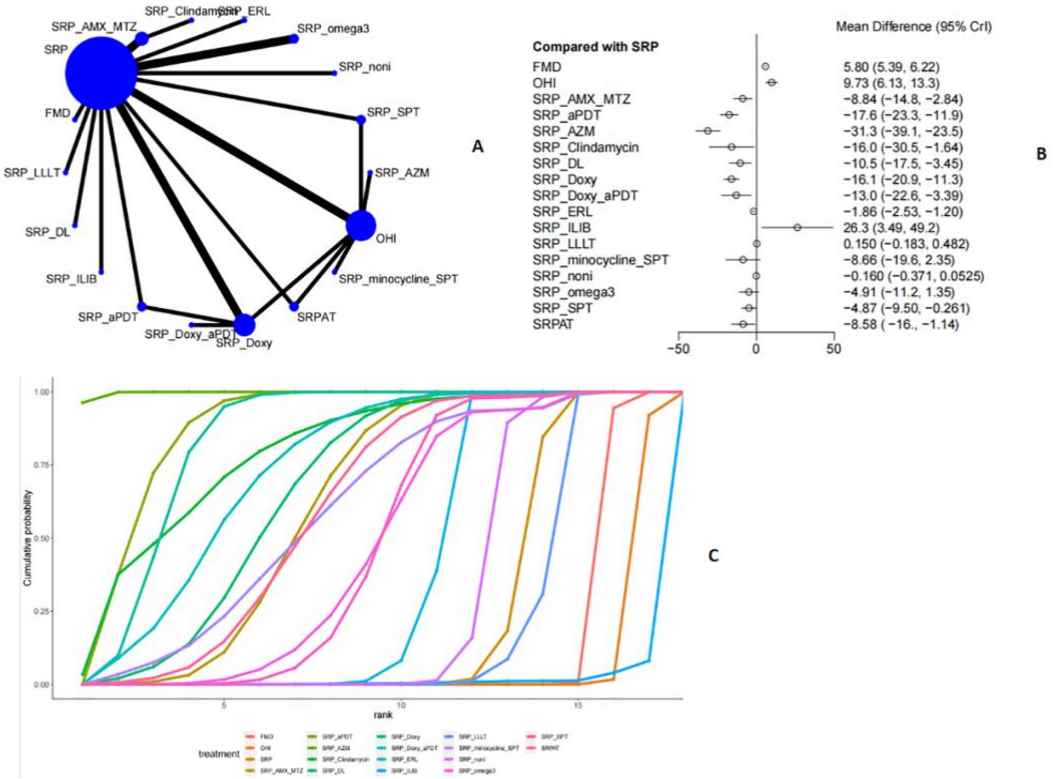

Figure S7: Local inconsistency test for BOP

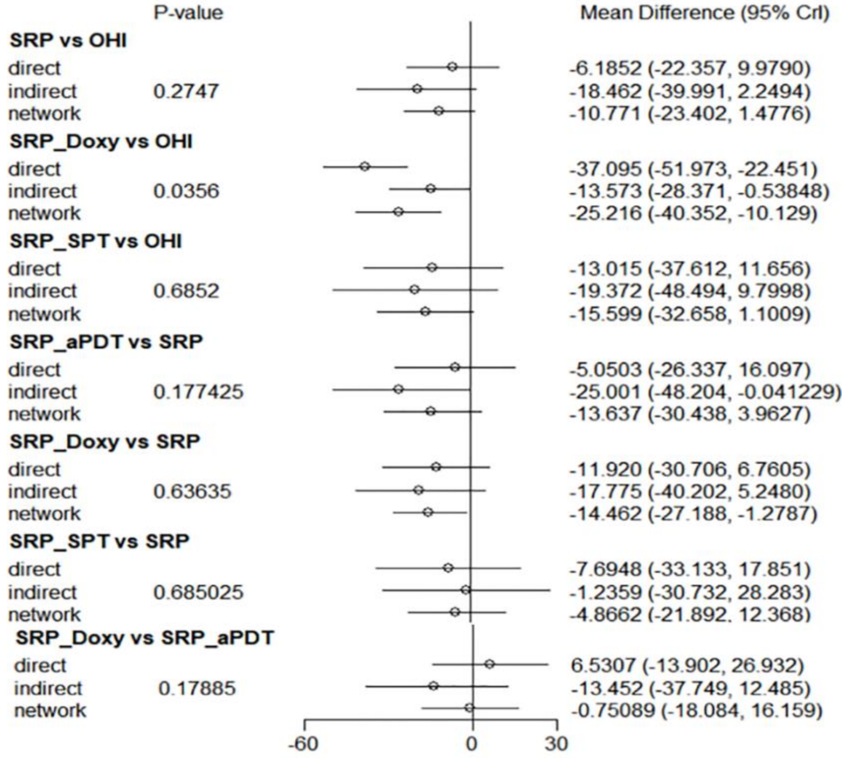

Figure S8: Funnel plot for BOP

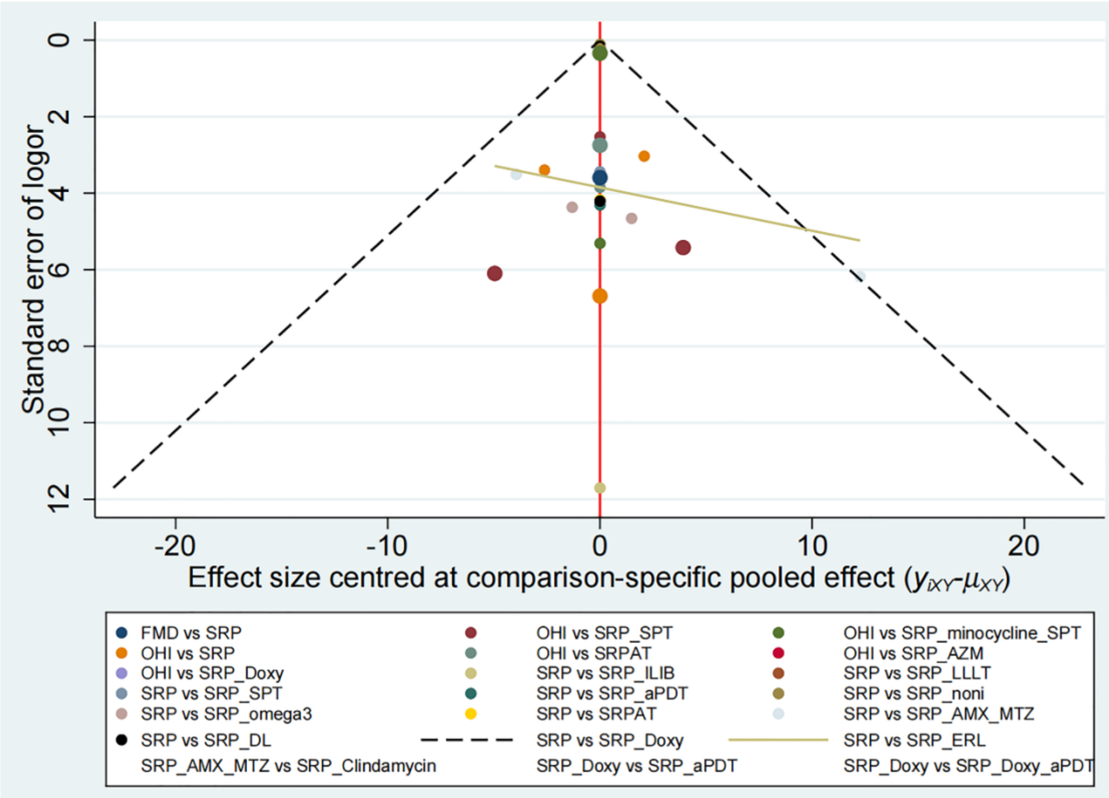

Figure S9: Meta-analysis of HbA1c%

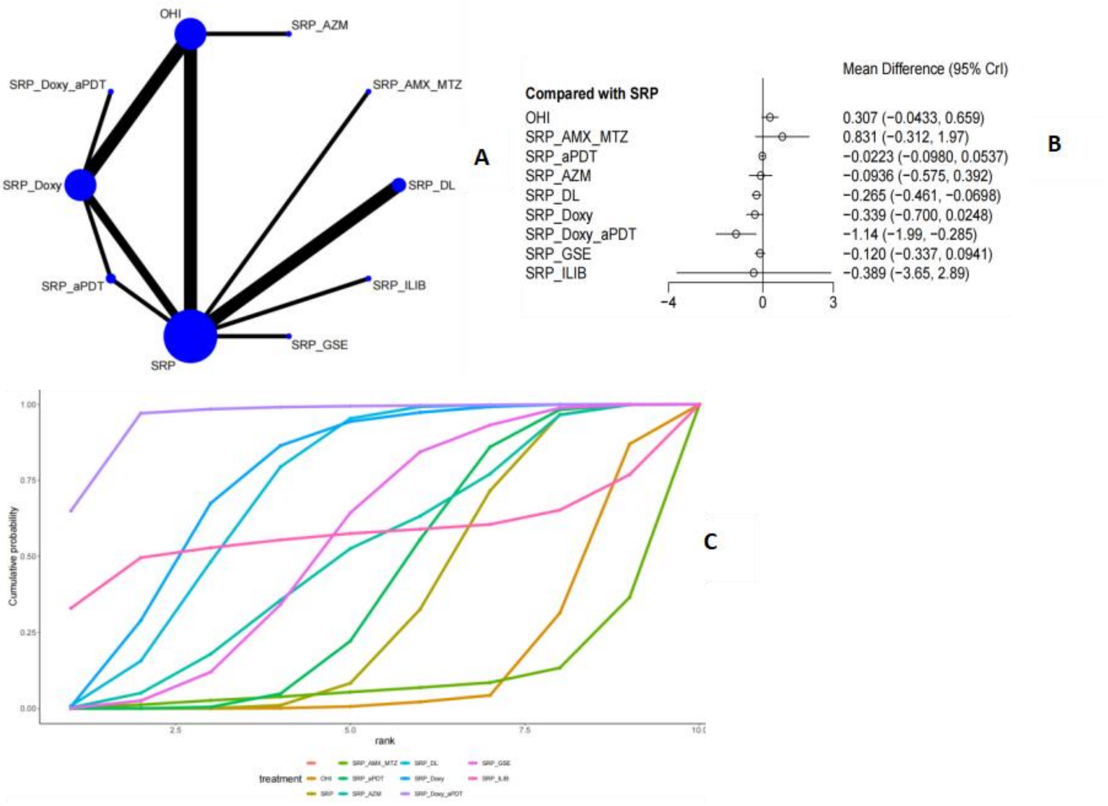

Figure S10: Local inconsistency test for HbA1c%

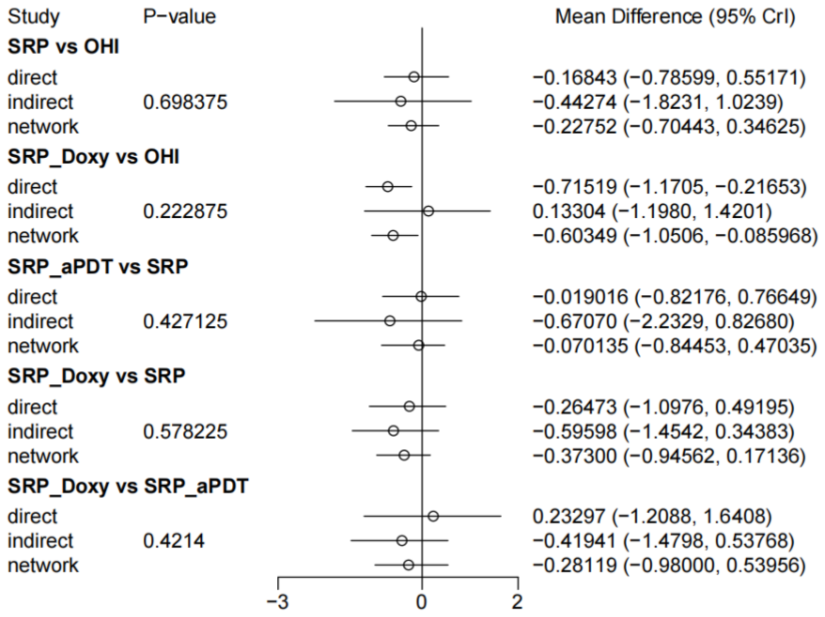

Figure S11: Funnel plot for HbA1c%

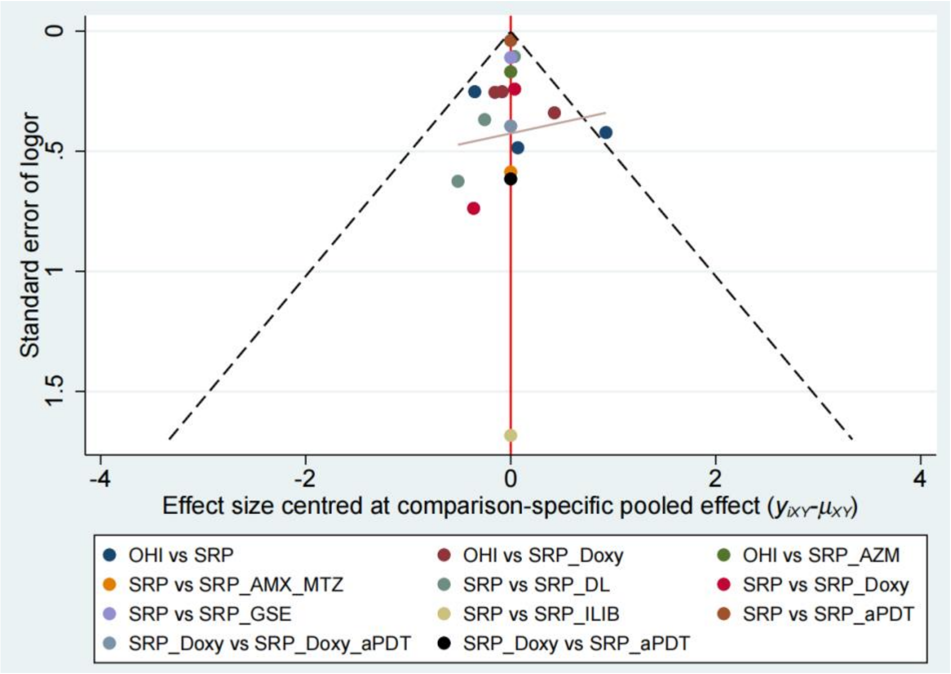

Figure S12: Meta-analysis of FBS

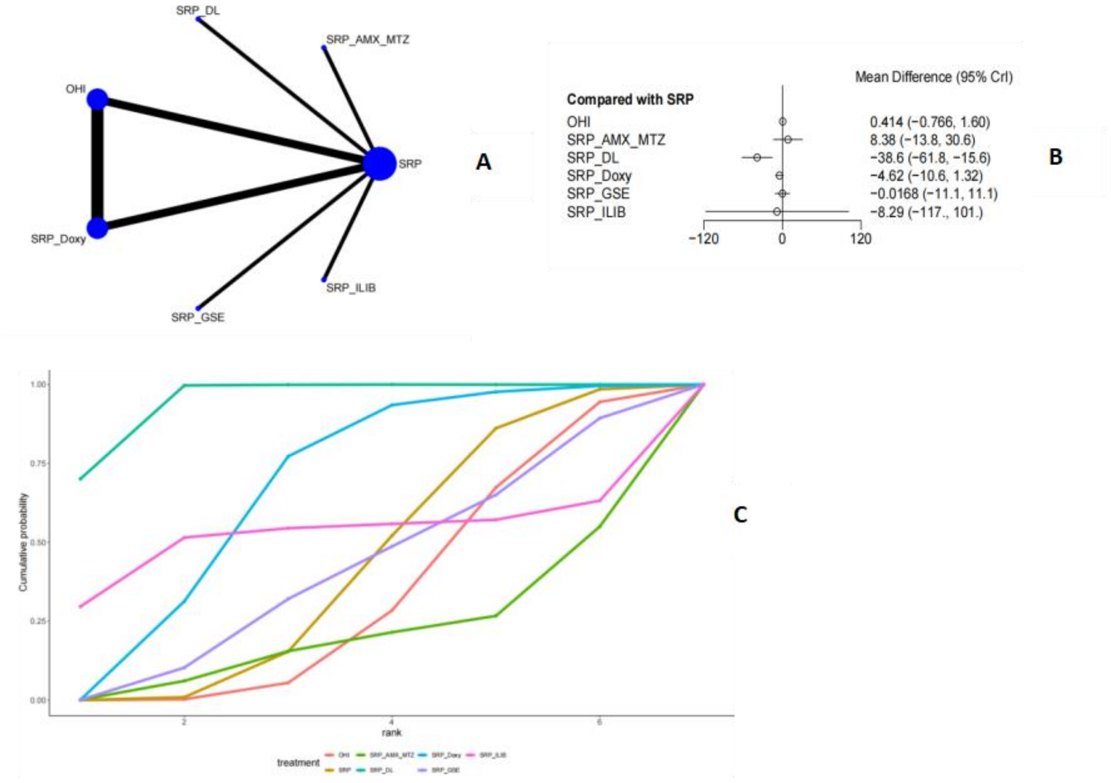

Figure S13: Local inconsistency test for FBS

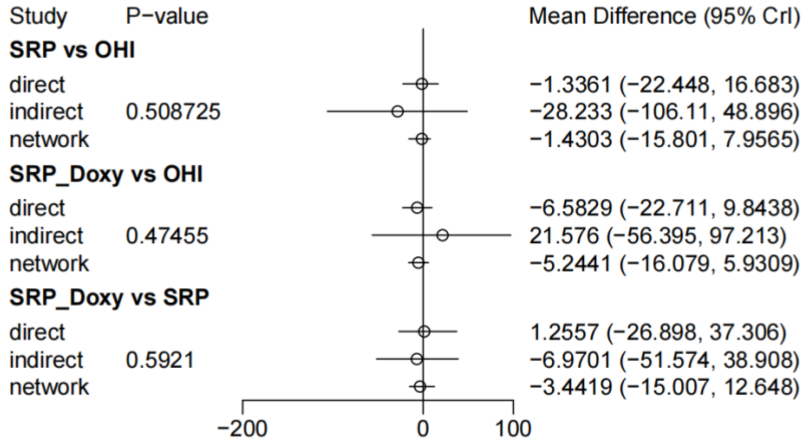

Figure S14: Funnel plot for HbA1c

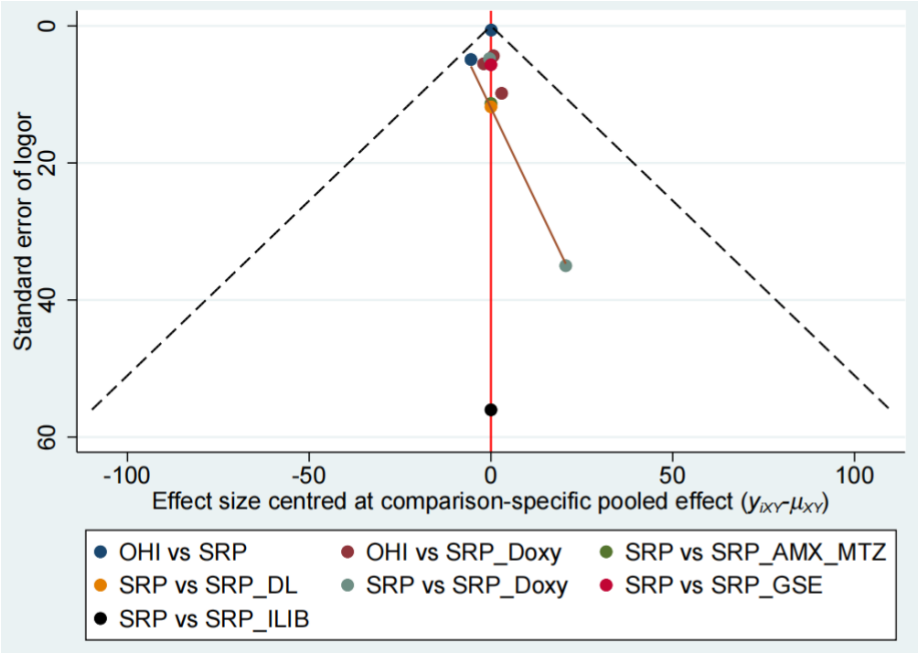

Standard error of logor
0
20
40
60
-100
-50
0
50
100
Effect size centred at comparison-specific pooled effect (y_{iXY}-μ_{XY})
OHI vs SRP
OHI vs SRP_Doxy
SRP vs SRP_AMX_MTZ
SRP vs SRP_DL
SRP vs SRP_Doxy
SRP vs SRP_GSE
SRP vs SRP_ILIB

Table S1 League table

League table for PD

MD 95%CrI																								
FMD																								
-0.32 (-0.53, -0.12)a	OHI																							
-0.1 (-0.3, 0.1)	0.22 (0.16, 0.29)a	SRP																						
0.73 (0.43, 1.03)a	1.05 (0.81, 1.29)a	0.83 (0.6, 1.06)a	SRP_AMX_MTZ																					
0.34 (0.07, 0.61)a	0.66 (0.47, 0.85)a	0.44 (0.26, 0.62)a	-0.39 (-0.68, -0.1)a	SRP_aPDT																				
0.68 (0.38, 0.98)a	1 (0.78, 1.22)a	0.78 (0.55, 1)a	-0.05 (-0.37, 0.27)	0.34 (0.05, 0.63)a	SRP_AZM																			
0.87 (0.43, 1.31)a	1.19 (0.79, 1.59)a	0.97 (0.57, 1.36)a	0.14 (-0.18, 0.46)	0.53 (0.09, 0.96)a	0.19 (-0.27, 0.64)	SRP_Clindamycin																		
0.4 (0.17, 0.63)a	0.72 (0.59, 0.86)a	0.5 (0.38, 0.62)a	-0.33 (-0.59, -0.07)a	0.06 (-0.15, 0.28)	-0.28 (-0.53, -0.02)a	-0.47 (-0.88, -0.05)a	SRP_DL																	
0.44 (0.21, 0.68)a	0.77 (0.64, 0.9)	0.54 (0.41, 0.68)	-0.28 (-0.55, -0.02)a	0.11 (-0.11, 0.32)	-0.23 (-0.48, 0.02)	-0.42 (-0.84, 0)	0.04 (-0.14, 0.22)	SRP_Doxy																
0.44 (-0.12, 0.99)	0.76 (0.24, 1.28)a	0.54 (0.01, 1.06)a	-0.29 (-0.86, 0.28)	0.1 (-0.45, 0.64)	-0.24 (-0.81, 0.32)	-0.43 (-1.08, 0.22)	0.03 (-0.5, 0.57)	-0.01 (-0.51, 0.49)	SRP_Doxy_aPDT															
1.05 (0.66, 1.44)a	1.37 (1.03, 1.71)a	1.15 (0.81, 1.48)a	0.32 (-0.08, 0.73)	0.71 (0.33, 1.09)a	0.37 (-0.03, 0.78)	0.18 (-0.33, 0.7)	0.65 (0.29, 1)a	0.61 (0.24, 0.96)a	0.62 (-0.01, 1.23)	SRP_ERL														
0.23 (-0.14, 0.59)	0.55 (0.24, 0.87)a	0.33 (0.02, 0.64)	-0.5 (-0.88, -0.11)a	-0.11 (-0.47, 0.25)	-0.45 (-0.83, -0.07)a	-0.64 (-1.14, -0.13)a	-0.17 (-0.5, 0.16)	-0.22 (-0.55, 0.12)	-0.2 (-0.81, 0.4)	-0.82 (-1.28, -0.36)a	SRP_ginger													
-0.11 (-0.42, 0.2)	0.21 (-0.03, 0.46)	-0.01 (-0.25, 0.23)	-0.84 (-1.17, -0.51)a	-0.45 (-0.75, -0.15)a	-0.79 (-1.11, -0.46)a	-0.98 (-1.44, -0.52)a	-0.51 (-0.78, -0.25)a	-0.56 (-0.83, -0.28)a	-0.54 (-1.12, 0.03)	-1.16 (-1.57, -0.75)a	-0.34 (-0.73, 0.05)	SRP_GSE												
0.4	0.73	0.5	-0.32	0.07	-0.27	-0.46	0	-0.04	-0.03	-0.65	0.17	0.51	SRP_I											

(-0.69, 1.5)	(-0.35, 1.81)	(-0.57, 1.58)	(-1.42, 0.77)	(-1.02, 1.16)	(-1.37, 0.83)	(-1.6, 0.68)	(-1.08, 1.09)	(-1.13, 1.04)	(-1.22, 1.16)	(-1.77, 0.48)	(-0.94, 1.29)	(-0.59, 1.62)	LIB											
-0.02 (-0.52, 0.48)	0.3 (-0.16, 0.76)	0.08 (-0.38, 0.54)	-0.75 (-1.26, -0.24)a	-0.36 (-0.85, 0.13)	-0.7 (-1.21, -0.19)a	-0.89 (-1.49, -0.28)a	-0.42 (-0.9, 0.05)	-0.46 (-0.94, 0.01)	-0.46 (-1.15, 0.24)	-1.07 (-1.64, -0.5)a	-0.25 (-0.8, 0.3)	0.09 (-0.43, 0.6)	-0.42 (-1.59, 0.74)	SRP_LLLT										
1.22 (0.8, 1.65)a	1.54 (1.16, 1.92)a	1.32 (0.95, 1.7)a	0.49 (0.05, 0.93)a	0.88 (0.47, 1.3)a	0.54 (0.11, 0.98)a	0.35 (-0.19, 0.9)	0.82 (0.43, 1.21)a	0.78 (0.38, 1.17)a	0.79 (0.15, 1.43)a	0.17 (-0.33, 0.68)	0.99 (0.5, 1.48)	1.33 (0.89, 1.78)	0.82 (-0.32, 1.95)	1.24 (0.65, 1.84)a	SRP_melatonin									
0.28 (-0.24, 0.79)	0.6 (0.13, 1.07)a	0.38 (-0.1, 0.86)	-0.45 (-0.98, 0.08)	-0.06 (-0.57, 0.45)	-0.4 (-0.92, 0.12)	-0.59 (-1.21, 0.03)	-0.12 (-0.62, 0.37)	-0.17 (-0.66, 0.32)	-0.16 (-0.86, 0.55)	-0.77 (-1.35, -0.19)a	0.05 (-0.52, 0.62)	0.39 (-0.14, 0.92)	-0.13 (-1.3, 1.05)	0.3 (-0.36, 0.96)	-0.94 (-1.55, -0.33)a	SRP_minocycline_SPT								
-0.61 (-0.91, -0.31)a	-0.29 (-0.53, -0.05)a	-0.51 (-0.74, -0.28)a	-1.34 (-1.67, -1.01)a	-0.95 (-1.24, -0.65)a	-1.29 (-1.61, -0.96)a	-1.48 (-1.94, -1.02)a	-1.01 (-1.27, -0.75)a	-1.06 (-1.32, -0.79)a	-1.05 (-1.62, -0.47)a	-1.66 (-2.07, -1.25)a	-0.84 (-1.23, -0.45)a	-0.5 (-0.83, -0.17)a	-1.01 (-2.11, 0.08)	-0.59 (-1.1, -0.07)a	-1.83 (-2.27, -1.39)a	-0.89 (-1.42, -0.36)a	SRP_noni							
-0.1 (-0.33, 0.13)	0.22 (0.09, 0.36)a	0 (-0.12, 0.12)	-0.83 (-1.09, -0.57)a	-0.44 (-0.65, -0.22)a	-0.78 (-1.03, -0.52)a	-0.97 (-1.38, -0.56)a	-0.5 (-0.67, -0.33)a	-0.55 (-0.72, -0.37)a	-0.53 (-1.07, 0)	-1.15 (-1.5, -0.79)a	-0.33 (-0.66, 0)	0.01 (-0.25, 0.27)	-0.5 (-1.59, 0.58)	-0.08 (-0.55, 0.4)	-1.32 (-1.71, -0.93)a	-0.38 (-0.87, 0.11)	0.51 (0.25, 0.77)a	SRP_omega3						
-0.01 (-0.22, 0.2)	0.31 (0.25, 0.38)a	0.09 (0.01, 0.17)a	-0.74 (-0.98, -0.49)a	-0.35 (-0.54, -0.15)a	-0.68 (-0.91, -0.46)a	-0.87 (-1.28, -0.47)a	-0.41 (-0.55, -0.26)a	-0.45 (-0.6, -0.31)a	-0.44 (-0.97, 0.08)	-1.06 (-1.4, -0.71)a	-0.24 (-0.56, 0.08)	0.1 (-0.15, 0.35)	-0.41 (-1.49, 0.67)	0.01 (-0.45, 0.48)	-1.23 (-1.61, -0.85)a	-0.29 (-0.76, 0.19)	0.6 (0.36, 0.85)a	0.09 (-0.05, 0.23)	SRP_SPT					
0.27 (-0.26, 0.8)	0.59 (0.1, 1.09)a	0.37 (-0.12, 0.86)	-0.46 (-1, 0.08)	-0.07 (-0.59, 0.45)	-0.41 (-0.95, 0.13)	-0.6 (-1.23, 0.03)	-0.13 (-0.64, 0.37)	-0.17 (-0.68, 0.33)	-0.16 (-0.88, 0.55)	-0.78 (-1.37, -0.18)a	0.04 (-0.54, 0.62)	0.38 (-0.17, 0.92)	-0.13 (-1.32, 1.05)	0.29 (-0.38, 0.96)	-0.95 (-1.57, -0.34)a	-0.01 (-0.69, 0.68)	0.88 (0.34, 1.42)a	0.37 (-0.14, 0.88)	0.28 (-0.22, 0.77)	SRP_Synbiotic				
2.54 (1.85, 3.23)a	2.87 (2.19, 3.53)a	2.64 (1.97, 3.31)a	1.81 (1.1, 2.52)a	2.2 (1.51, 2.89)a	1.87 (1.16, 2.57)a	1.68 (0.9, 2.44)a	2.14 (1.46, 2.81)a	2.1 (1.41, 2.78)a	2.11 (1.26, 2.95)a	1.49 (0.75, 2.24)a	2.31 (1.58, 3.04)a	2.65 (1.94, 3.36)a	2.14 (0.87, 3.4)a	2.56 (1.75, 3.37)a	1.32 (0.55, 2.08)a	2.27 (1.44, 3.08)a	3.15 (2.44, 3.86)a	2.64 (1.96, 3.32)a	2.55 (1.88, 3.22)a	2.27 (1.44, 3.1)a	SRP_SZ			
1 (0.51, 1.49)a	1.32 (0.87, 1.77)a	1.1 (0.65, 1.55)a	0.27 (-0.23, 0.78)	0.66 (0.18, 1.14)a	0.32 (-0.18, 0.82)	0.13 (-0.46, 0.73)	0.6 (0.13, 1.06)a	0.56 (0.09, 1.02)a	0.57 (-0.12, 1.25)	-0.05 (-0.61, 0.5)	0.77 (0.23, 1.31)a	1.11 (0.61, 1.61)a	0.6 (-0.57, 1.76)	1.02 (0.38, 1.66)a	-0.22 (-0.81, 0.36)	0.72 (0.06, 1.38)a	1.61 (1.11, 2.12)a	1.1 (0.64, 1.56)a	1.01 (0.55, 1.46)a	0.73 (0.07, 1.39)a	-1.54 (-2.34, -0.74)a	SRP_vitaminD3		
0.93 (0.44, 1.42)a	1.25 (0.8, 1.71)a	1.03 (0.58, 1.48)a	0.2 (-0.3, 0.71)	0.59 (0.1, 1.08)a	0.25 (-0.25, 0.76)	0.06 (-0.54, 0.66)	0.53 (0.06, 1)a	0.49 (0.02, 0.96)a	0.5 (-0.19, 1.19)	-0.12 (-0.68, 0.44)	0.7 (0.15, 1.25)a	1.04 (0.53, 1.55)a	0.53 (-0.64, 1.69)	0.95 (0.31, 1.6)a	-0.29 (-0.88, 0.3)	0.65 (0, 1.31)a	1.54 (1.03, 2.05)a	1.03 (0.56, 1.5)a	0.94 (0.48, 1.4)a	0.66 (-0.01, 1.33)	-1.61 (-2.42, -0.81)a	-0.07 (-0.71, 0.57)	SRP_ZLN	
-0.07 (-0.3, 0.15)	0.25 (0.14, 0.35)a	0.03 (-0.08, 0.13)	-0.8 (-1.06, -0.55)a	-0.41 (-0.62, -0.21)a	-0.75 (-0.99, -0.51)a	-0.94 (-1.35, -0.53)a	-0.48 (-0.64, -0.32)a	-0.52 (-0.68, -0.36)a	-0.51 (-1.04, 0.02)	-1.12 (-1.47, -0.77)a	-0.3 (-0.63, 0.02)	0.04 (-0.22, 0.29)	-0.48 (-1.56, 0.6)	-0.05 (-0.52, 0.42)	-1.3 (-1.69, -0.91)a	-0.35 (-0.84, 0.13)	0.54 (0.28, 0.79)a	0.03 (-0.13, 0.18)	-0.07 (-0.19, 0.05)	-0.35 (-0.84, 0.15)	-2.62 (-3.29, -1.94)a	-1.08 (-1.53, -0.62)a	-1.01 (-1.47, -0.54)a	SRPAT
a means P＜0.05																								

League table for CAL

MD 95%CrI																				
FMD																				
-0.49 (-1.15, 0.18)	OHI																			
-0.3 (-0.96, 0.36)	0.19 (0.11, 0.27)a	SRP																		
0.2 (-0.58, 0.98)	0.69 (0.27, 1.11)a	0.5 (0.09, 0.91)a	SRP_AMX_MTZ																	
0.2 (-0.5, 0.89)	0.69 (0.46, 0.91)a	0.5 (0.28, 0.71)a	0 (-0.47, 0.46)	SRP_aPDT																
0.34 (-0.36, 1.02)	0.82 (0.61, 1.04)a	0.63 (0.43, 0.84)a	0.13 (-0.33, 0.59)	0.14 (-0.16, 0.43)	SRP_DL															
0.07 (-0.62, 0.76)	0.56 (0.37, 0.75)a	0.37 (0.17, 0.56)a	-0.13 (-0.59, 0.32)	-0.13 (-0.4, 0.15)	-0.27 (-0.55, 0.01)	SRP_Doxy														
0.15 (-0.77, 1.07)	0.64 (0.01, 1.27)	0.45 (-0.18, 1.09)	-0.05 (-0.81, 0.7)	-0.05 (-0.71, 0.62)	-0.18 (-0.85, 0.48)	0.08 (-0.52, 0.69)	SRP_Doxy_aPDT													
0.4 (-0.38, 1.18)	0.89 (0.46, 1.32)a	0.7 (0.28, 1.12)a	0.2 (-0.39, 0.79)	0.2 (-0.27, 0.68)	0.07 (-0.4, 0.53)	0.33 (-0.13, 0.8)	0.25 (-0.51, 1.01)	SRP_ERL												
0.13 (-0.58, 0.84)	0.62 (0.35, 0.89)a	0.43 (0.17, 0.69)a	-0.07 (-0.56, 0.41)	-0.07 (-0.4, 0.27)	-0.2 (-0.53, 0.12)	0.06 (-0.26, 0.39)	-0.02 (-0.7, 0.66)	-0.27 (-0.77, 0.23)	SRP_ginger											
-0.4 (-1.11, 0.31)	0.09 (-0.2, 0.38)	-0.1 (-0.38, 0.18)	-0.6 (-1.1, -0.1)a	-0.6 (-0.95, -0.24)a	-0.73 (-1.08, -0.39)a	-0.47 (-0.81, -0.13)a	-0.55 (-1.24, 0.14)	-0.8 (-1.31, -0.29)a	-0.53 (-0.91, -0.15)a	SRP_GSE										
0.2 (-1.63, 2.03)	0.69 (-1.02, 2.4)	0.5 (-1.21, 2.2)	0 (-1.75, 1.75)	0 (-1.72, 1.72)	-0.14 (-1.85, 1.59)	0.13 (-1.58, 1.85)	0.05 (-1.77, 1.87)	-0.2 (-1.95, 1.55)	0.07 (-1.66, 1.8)	0.6 (-1.13, 2.33)	SRP_ILIB									
-0.29 (-1.05, 0.47)	0.2 (-0.19, 0.59)	0.01 (-0.37, 0.39)	-0.49 (-1.05, 0.07)	-0.49 (-0.92, -0.05)a	-0.63 (-1.05, -0.19)a	-0.36 (-0.79, 0.07)	-0.44 (-1.18, 0.3)	-0.69 (-1.26, -0.12)a	-0.42 (-0.88, 0.04)	0.11 (-0.36, 0.58)	-0.49 (-2.24, 1.25)	SRP_LLLT								
0.93 (0.21,	1.42 (1.11,	1.23 (0.93,	0.73 (0.22, 1.24)a	0.73 (0.36, 1.1)a	0.6 (0.23, 0.96)a	0.86 (0.5, 1.22)a	0.78 (0.08,	0.53 (0.01, 1.05)a	0.8 (0.4, 1.2)a	1.33 (0.92,	0.73 (-1, 2.47)	1.22 (0.73,	SRP_melatonin							

1.66)a	1.73)a	1.53)a					1.48)a			1.74)a		1.71)a								
-0.23 (-0.94, 0.48)	0.26 (-0.01, 0.53)	0.07 (-0.19, 0.33)	-0.43 (-0.92, 0.06)	-0.43 (-0.76, -0.09)a	-0.56 (-0.89, -0.23)a	-0.3 (-0.62, 0.03)	-0.38 (-1.07, 0.3)	-0.63 (-1.12, -0.13)a	-0.36 (-0.73, 0.01)	0.17 (-0.21, 0.55)	-0.43 (-2.16, 1.29)	0.06 (-0.4, 0.52)	-1.16 (-1.56, -0.76)	SRP_noni						
-0.13 (-0.81, 0.54)	0.35 (0.18, 0.53)a	0.16 (0, 0.33)a	-0.34 (-0.78, 0.11)	-0.33 (-0.6, -0.06)a	-0.47 (-0.73, -0.21)a	-0.2 (-0.46, 0.05)	-0.29 (-0.94, 0.37)	-0.54 (-0.99, -0.08)a	-0.27 (-0.57, 0.04)	0.26 (-0.06, 0.58)	-0.33 (-2.05, 1.38)	0.16 (-0.26, 0.57)	-1.07 (-1.41, -0.72)	0.09 (-0.21, 0.4)	SRP_omega3					
-0.26 (-0.93, 0.4)	0.23 (0.13, 0.32)a	0.04 (-0.06, 0.14)	-0.47 (-0.89, -0.04)a	-0.46 (-0.7, -0.22)a	-0.6 (-0.82, -0.37)a	-0.33 (-0.54, -0.13)a	-0.41 (-1.05, 0.22)	-0.66 (-1.1, -0.23)a	-0.39 (-0.67, -0.11)a	0.14 (-0.16, 0.43)	-0.46 (-2.17, 1.25)	0.03 (-0.37, 0.42)	-1.19 (-1.51, -0.88)	-0.03 (-0.31, 0.24)	-0.13 (-0.32, 0.06)	SRP_SPT				
0.1 (-0.6, 0.8)	0.59 (0.33, 0.85)a	0.4 (0.15, 0.65)a	-0.1 (-0.58, 0.38)	-0.1 (-0.42, 0.23)	-0.23 (-0.55, 0.09)	0.03 (-0.28, 0.35)	-0.05 (-0.73, 0.63)	-0.3 (-0.79, 0.19)	-0.03 (-0.39, 0.33)	0.5 (0.13, 0.87)a	-0.1 (-1.82, 1.62)	0.39 (-0.06, 0.84)	-0.83 (-1.22, -0.44)	0.33 (-0.03, 0.69)	0.24 (-0.06, 0.53)	0.36 (0.09, 0.63)	SRP_Synbiotic			
1.98 (1.07, 2.89)a	2.47 (1.84, 3.1)a	2.28 (1.66, 2.9)a	1.78 (1.03, 2.52)a	1.78 (1.13, 2.44)a	1.64 (0.99, 2.3)a	1.91 (1.26, 2.56)a	1.83 (0.94, 2.71)a	1.58 (0.83, 2.33)a	1.85 (1.17, 2.52)a	2.38 (1.7, 3.06)a	1.78 (-0.04, 3.6)	2.27 (1.54, 3)a	1.05 (0.36, 1.74)a	2.21 (1.54, 2.88)a	2.11 (1.47, 2.76)a	2.24 (1.61, 2.87)a	1.88 (1.21, 2.55)a	SRP_SZ		
0.88 (0.13, 1.64)a	1.37 (0.99, 1.75)a	1.18 (0.81, 1.56)a	0.68 (0.12, 1.23)a	0.68 (0.25, 1.12)a	0.55 (0.12, 0.97)a	0.81 (0.39, 1.23)a	0.73 (-0.01, 1.47)	0.48 (-0.08, 1.04)	0.75 (0.3, 1.21)a	1.28 (0.81, 1.74)a	0.68 (-1.06, 2.43)	1.17 (0.64, 1.71)a	-0.05 (-0.53, 0.43)	1.11 (0.66, 1.57)a	1.02 (0.61, 1.42)a	1.14 (0.76, 1.53)	0.78 (0.33, 1.23)a	-1.1 (-1.82, -0.37)a	SRP_vitaminD3	
0.73 (-0.08, 1.53)	1.22 (0.75, 1.69)a	1.03 (0.57, 1.49)a	0.53 (-0.09, 1.15)	0.53 (0.02, 1.04)a	0.39 (-0.11, 0.9)	0.66 (0.16, 1.16)a	0.58 (-0.21, 1.37)	0.33 (-0.3, 0.96)	0.6 (0.07, 1.13)a	1.13 (0.59, 1.67)a	0.53 (-1.24, 2.3)	1.02 (0.42, 1.62)a	-0.2 (-0.75, 0.35)	0.96 (0.43, 1.49)a	0.86 (0.38, 1.35)a	0.99 (0.52, 1.47)a	0.63 (0.1, 1.15)a	-1.25 (-2.02, -0.48)a	-0.15 (-0.74, 0.44)	SRP_ZLN
a means P＜0.05																				

League table for BOP

MD 95%CrI																	
FMD																	
-3.92 (-7.56, -0.31)a	OHI																
5.8 (5.39, 6.22)a	9.73 (6.13, 13.33)a	SRP															
14.64 (8.62, 20.6)a	18.57 (11.6, 25.5)a	8.84 (2.84, 14.79)a	SRP_AMX_MTZ														
23.38 (17.69, 29.09)a	27.3 (21.24, 33.41)a	17.58 (11.91, 23.28)a	8.74 (0.55, 17.03)a	SRP_aPDT													
37.07 (29.24, 44.91)a	41 (34.07, 47.94)a	31.27 (23.46, 39.1)a	22.42 (12.65, 32.3)a	13.69 (4.44, 22.92)a	SRP_AZM												

21.81 (7.43, 36.27)a	25.74 (10.94, 40.63)a	16.01 (1.64, 30.47)a	7.18 (-5.89, 20.33)	-1.58 (-17.02, 13.99)	-15.25 (-31.57, 1.15)	SRP_Clindamycin											
16.29 (9.25, 23.33)a	20.22 (12.3, 28.09)a	10.49 (3.45, 17.52)a	1.66 (-7.59, 10.88)	-7.09 (-16.12, 1.9)	-20.78 (-31.3, -10.29)a	-5.54 (-21.61, 10.47)	SRP_DL										
21.91 (17.04, 26.77)a	25.83 (20.88, 30.82)a	16.1 (11.25, 20.95)a	7.26 (-0.34, 14.95)	-1.49 (-6.21, 3.26)	-15.16 (-23.73, -6.67)a	0.07 (-15.17, 15.26)	5.62 (-2.89, 14.17)	SRP_Doxy									
18.83 (9.18, 28.4)a	22.75 (13.07, 32.36)a	13.03 (3.39, 22.6)a	4.19 (-7.1, 15.45)	-4.56 (-14.05, 4.93)	-18.23 (-30.12, -6.37)a	-3 (-20.29, 14.31)	2.52 (-9.35, 14.41)	-3.07 (-11.36, 5.12)	SRP_Doxy_aPDT								
7.66 (6.87, 8.45)a	11.59 (7.93, 15.25)a	1.86 (1.2, 2.53)a	-6.98 (-12.97, -0.94)a	-15.72 (-21.46, -10.01)a	-29.41 (-37.26, -21.56)a	-14.15 (-28.62, 0.22)	-8.63 (-15.68, -1.56)a	-14.25 (-19.12, -9.35)a	-11.18 (-20.77, -1.5)a	SRP_ERL							
-20.5 (-43.4, 2.34)	-16.56 (-39.72, 6.48)	-26.31 (-49.22, -3.49)a	-35.13 (-58.84, -11.56)a	-43.88 (-67.54, -20.35)a	-57.55 (-81.77, -33.49)a	-42.33 (-69.54, -15.25)a	-36.79 (-60.7, -12.89)a	-42.39 (-65.9, -19.13)a	-39.32 (-64.13, -14.57)a	-28.15 (-51.08, -5.32)a	SRP_ILIB						
5.65 (5.12, 6.18)a	9.58 (5.96, 13.19)a	-0.15 (-0.48, 0.18)	-8.99 (-14.95, -2.98)a	-17.73 (-23.43, -12.06)a	-31.42 (-39.26, -23.59)a	-16.16 (-30.6, -1.78)a	-10.64 (-17.67, -3.6)a	-16.25 (-21.11, -11.4)a	-13.19 (-22.75, -3.53)a	-2.01 (-2.76, -1.27)a	26.16 (3.33, 49.07)a	SRP_LLLT					
14.46 (3.45, 25.44)a	18.37 (7.98, 28.77)a	8.66 (-2.35, 19.65)	-0.17 (-12.69, 12.35)	-8.93 (-20.95, 3.13)	-22.64 (-35.05, -10.09)a	-7.35 (-25.51, 10.65)	-1.85 (-14.8, 11.22)	-7.45 (-19.04, 4.11)	-4.38 (-18.5, 9.89)	6.79 (-4.22, 17.81)	34.92 (9.59, 60.33)a	8.8 (-2.21, 19.79)	SRP_minocycline_SPT				
5.96 (5.49, 6.43)a	9.89 (6.28, 13.5)a	0.16 (-0.05, 0.37)	-8.68 (-14.63, -2.67)a	-17.42 (-23.13, -11.74)a	-31.11 (-38.95, -23.29)a	-15.85 (-30.31, -1.48)a	-10.33 (-17.36, -3.29)a	-15.94 (-20.8, -11.09)a	-12.87 (-22.45, -3.22)a	-1.7 (-2.4, -1)a	26.46 (3.65, 49.38)a	0.31 (-0.09, 0.7)a	-8.49 (-19.48, 2.51)	SRP_noni			
10.71 (4.43, 16.99)a	14.64 (7.43, 21.89)a	4.91 (-1.35, 11.17)	-3.91 (-12.54, 4.76)	-12.65 (-21.1, -4.26)a	-26.35 (-36.32, -16.37)a	-11.09 (-26.87, 4.63)	-5.56 (-14.96, 3.84)	-11.18 (-19.06, -3.31)a	-8.11 (-19.5, 3.34)	3.05 (-3.26, 9.36)	31.21 (7.5, 54.96)a	5.06 (-1.21, 11.33)	-3.73 (-16.42, 8.91)	4.75 (-1.51, 11.01)	SRP_omega3		
10.67 (6.05, 15.32)a	14.6 (10.41, 18.78)a	4.87 (0.26, 9.5)a	-3.96 (-11.49, 3.6)	-12.7 (-19.7, -5.75)a	-26.41 (-34.47, -18.29)a	-11.15 (-26.32, 3.94)	-5.63 (-14.02, 2.81)	-11.23 (-17.37, -5.12)a	-8.15 (-18.46, 2.2)	3.01 (-1.65, 7.7)	31.16 (7.94, 54.51)a	5.02 (0.4, 9.66)a	-3.78 (-14.99, 7.46)	4.71 (0.1, 9.35)a	-0.05 (-7.8, 7.77)	SRP_SPT	
14.38 (6.93, 21.8)a	18.29 (11.08, 25.53)a	8.58 (1.14, 16)a	-0.26 (-9.78, 9.31)	-8.99 (-18.1, 0.08)	-22.69 (-32.75, -12.64)a	-7.45 (-23.68, 8.76)	-1.92 (-12.17, 8.38)	-7.52 (-16.03, 0.94)	-4.44 (-16.31, 7.42)	6.72 (-0.76, 14.17)	34.86 (10.92, 58.94)a	8.73 (1.29, 16.15)a	-0.09 (-12.76, 12.56)	8.42 (0.98, 15.84)a	3.66 (-6.06, 13.32)	3.71 (-4.43, 11.84)	SRPAT

a means $P<0.05$

League table for HbA1c%

MD 95%CrI									
OHI									
0.31 (-0.04, 0.66)	SRP								
-0.52 (-1.72, 0.68)	-0.83 (-1.97, 0.31)	SRP_AMX_MTZ							
0.33 (-0.03, 0.69)	0.02 (-0.05, 0.1)	0.85 (-0.29, 2)	SRP_aPDT						
0.4 (0.07, 0.73)a	0.09 (-0.39, 0.58)	0.92 (-0.32, 2.17)	0.07 (-0.42, 0.56)	SRP_AZM					
0.57 (0.17, 0.97)a	0.27 (0.07, 0.46)a	1.1 (-0.07, 2.26)	0.24 (0.03, 0.45)a	0.17 (-0.35, 0.69)	SRP_DL				
0.65 (0.35, 0.94)a	0.34 (-0.02, 0.7)	1.17 (-0.03, 2.37)	0.32 (-0.05, 0.68)	0.25 (-0.2, 0.69)	0.07 (-0.34, 0.48)	SRP_Doxy			
1.45 (0.61, 2.28)a	1.14 (0.28, 1.99)a	1.97 (0.54, 3.4)a	1.12 (0.26, 1.97)a	1.05 (0.15, 1.94)a	0.87 (0, 1.75)a	0.8 (0.02, 1.57)a	SRP_Doxy_aPDT		
0.43 (0.02, 0.84)a	0.12 (-0.09, 0.34)	0.95 (-0.22, 2.12)	0.1 (-0.13, 0.33)	0.03 (-0.5, 0.56)	-0.15 (-0.44, 0.15)	-0.22 (-0.64, 0.2)	-1.02 (-1.9, -0.14)a	SRP_GSE	
0.7 (-2.61, 3.98)	0.39 (-2.89, 3.65)	1.22 (-2.26, 4.68)	0.37 (-2.92, 3.63)	0.3 (-3.02, 3.6)	0.12 (-3.16, 3.4)	0.05 (-3.25, 3.33)	-0.75 (-4.13, 2.63)	0.27 (-3.02, 3.54)	SRP_ILIB
a means P＜0.05									

League table for FBS

MD 95%CrI						
OHI						
0.42 (-0.77, 1.6)	SRP					
-7.99 (-30.3, 14.17)	-8.39 (-30.69, 13.73)	SRP_AMX_MTZ				
38.98 (15.63, 62.13)a	38.56 (15.23, 61.68)a	46.95 (14.96, 79.21)a	SRP_DL			

5.02 (-0.86, 10.88)	4.6 (-1.32, 10.53)	13.04 (-9.88, 35.94)	-33.96 (-57.9, -9.94)a	SRP_Doxy		
0.42 (-10.79, 11.59)	0.01 (-11.14, 11.08)	8.45 (-16.39, 33.22)	-38.52 (-64.19, -12.8)a	-4.59 (-17.21, 7.96)	SRP_GSE	
8.66 (-100.24, 117.85)	8.26 (-100.73, 117.39)	16.73 (-94.85, 128.34)	-30.21 (-142.07, 81.27)a	3.73 (-105.62, 113.08)	8.26 (-101.43, 117.92)	SRP_ILIB
a means P＜0.05						
